# Helicase protein DDX11 as a novel antiviral factor promoting RIG-I-MAVS-mediated signaling pathway

**DOI:** 10.1128/mbio.02028-24

**Published:** 2024-10-29

**Authors:** Jiyu Zhang, Liaoyuan Zhang, Dakai Liu, Hongyan Shi, Xin Zhang, Jianfei Chen, Xiaoman Yang, Miaomiao Zeng, Jialin Zhang, Tingshuai Feng, Xiaoyuan Zhu, Zhaoyang Jing, Zhaoyang Ji, Da Shi, Li Feng

**Affiliations:** 1 State Key Laboratory for Animal Disease Control and Prevention, Harbin Veterinary Research Institute, Chinese Academy of Agricultural Sciences, Harbin, China; Fondazione Biotecnopolo di Siena, Siena, Italy

**Keywords:** DDX11, RIG-I, MAVS, IFN

## Abstract

Type Ι interferon (IFN) production mediated by retinoic acid-inducible gene 1 (RIG-I) and mitochondrial antiviral signaling protein (MAVS) is essential for antiviral innate immune responses. Here, we report the identification of a novel co-sensor for cytosolic nucleic acids: DEAD/H-box helicase 11 (DDX11), a member of the DExD/H (Asp-Glu-x-Asp/His)-box helicase family. Knockdown or knockout of DDX11 attenuated the ability of cells to increase IFN-β, IFN-stimulated gene 56, and C-X-C motif chemokine ligand 10 in response to SeV and poly (I:C) by blocking the activation of TANK-binding kinase 1 and IFN regulatory factor 3. Nucleic acid sensing by DDX11 was independent of the stimulator of IFN genes but was dependent on RIG-I and MAVS. DDX11 regulated RIG-I-MAVS-mediated IFN signaling by specifically interacting with nucleic acid, RIG-I, and MAVS to enhance RIG-I-double-strand RNA and RIG-I-MAVS binding affinity. Overall, our results identified a critical role for DDX11 in the innate immune response and provided molecular insights into the mechanisms by which DDX11 recognized cytosolic nucleic acid and interacted with RIG-Ι and MAVS for potent IFN signaling and antiviral immunity.

**IMPORTANCE:**

Innate immunity is the first and most rapid host defense against virus infection. Recognition of viral RNA by the retinoic acid-inducible gene 1 (RIG-I)-like receptors (RLRs) initiates innate antiviral immune responses. How the binding of viral RNA to and activation of the RLRs are regulated remains enigmatic. In this study, we identified DEAD/H-box helicase 11 (DDX11) as a positive regulator of the RIG-I-mitochondrial antiviral signaling protein (MAVS)-mediated signaling pathways. Mechanistically, we demonstrated that DDX11 bound to viral RNA, interacted with RIG-I, and promoted their binding to viral RNA. DDX11 also promoted the interaction between RIG-I and MAVS and activation of RIG-I-MAVS signaling. Overall, our results elucidate the role of DDX11 in RIG-I-MAVS-dependent signaling pathways and may shed light on innate immune gene regulation.

## INTRODUCTION

The innate immune system serves as the initial defense mechanism against microbial infections. Upon encountering pathogens, conserved microbial components known as pathogen-associated molecular patterns (PAMPs) are recognized by cellular pattern recognition receptors (PRRs). This recognition triggers a series of intracellular signal transduction pathways that ultimately result in the upregulation of type I interferon (IFN), pro-inflammatory cytokines, and an array of effector genes. The downstream effector proteins are involved in innate immune and inflammatory responses by impeding microbial replication and facilitating the clearance of infected cells. Consequently, the host adaptive immune response is enhanced, aiding in the elimination of infected pathogens (1–4).

Over the preceding decade, the use of genomic and genetic methodologies has elucidated three primary categories of innate immune receptors responsible for detecting microbial entities. The retinoic acid-inducible gene 1 (RIG-Ι)-like receptors (RLRs), including RIG-Ι, can sense intracellular viral RNA (5–7). NOD-like receptors can sense intracellular microbes (8, 9). Toll-like receptors (TLRs) represent a subclass of membrane-bound receptors that are adept at recognizing extracellular microbial agents, subsequently instigating signaling cascades aimed at combating pathogens (10–12). In addition to the well-known nucleic acid sensors, a growing number of DExD/H-box helicases have been identified to regulate RLR-mediated IFN induction in recent years. These helicases serve as supplementary receptors for viral nucleic acids or as mediators of downstream signaling pathways, thus increasing the complexity of innate immune response regulation. For instance, an RNA helicase complex comprising DEAD-box 1 (DDX1), DDX21, and DEAH-box helicase 36 induces IFN production via Toll-interleukin receptor-domain-containing adapter-inducing interferon-β-dependent signaling pathways (13). DDX24 negatively modulates RLR-dependent innate immune responses by competing with RIG-I for viral RNA binding and by suppressing IFN regulatory factor 7 (IRF7) phosphorylation via its interaction with receptor-interacting protein kinase 1 (14). DHX29 as an RNA co-sensor plays a role in the regulation of melanoma differentiation-associated protein 5 (MDA5)-mediated antiviral signaling by augmenting the affinity of MDA5 for double-strand RNA (dsRNA) (15). DDX60 and DDX3 interact with DDX58/MDA5, enhancing its capacity to recognize dsRNA and subsequently increasing the production of downstream IFN (16, 17). Finally, DDX41 helicase functions as a DNA sensor to initiate IFN production by targeting the stimulator of interferon genes-TANK-binding kinase 1 complex (18). These recent investigations underscore the essential functions of DExD/H-box helicases in eliciting antiviral immune responses. Nevertheless, the involvement of additional helicases in the detection of viral RNA or DNA remains uncertain.

In 2017, a newly identified bat-HKU2-like porcine alphacoronavirus, designated as swine acute diarrhea syndrome-coronavirus (SADS-CoV), was initially detected in Guangdong province, China. SADS-CoV induces severe watery diarrhea in neonatal piglets, leading to a high mortality rate approaching 90% (19). SADS-CoV has broad species cell tropism and grows efficiently in primary human cells, highlighting a potential risk of its transmission across host species barriers (20). These properties make SADS-CoV an ideal RNA virus for laboratory research.

RIG-I expression is typically low in many cell types and is induced after viral infection. Genetic investigations have elucidated the pivotal functions of RIG-I in the innate immune response against various RNA viruses (21). In addition, it has been demonstrated that RIG-I was responsible for sensing infection of vesicular stomatitis virus (VSV), Sendai virus (SeV), and SADS-CoV (5, 22–24). Most importantly, the expression of RIG-I in uninfected cells is low. It remains unclear whether (and if so, how) the binding of viral RNA to RIG-Ι is regulated for efficient sensing of viral RNA.

In the present study, DDX11 was identified as a significant modulator of RIG-Ι-MAVS-mediated signaling pathways. Knockdown or knockout of DDX11 suppressed the transcriptional activation of downstream antiviral genes and dampened the innate antiviral response induced by SeV and poly(I:C). Mechanistically, we demonstrated that DDX11 bound to viral RNA, interacted with RIG-Ι, and promoted their binding to viral RNA. DDX11 also promoted the interaction between RIG-Ι and MAVS, and activation of RIG-Ι-MAVS signaling. The collective findings revealed the previously unrecognized role of DDX11 in antiviral immunity and provided valuable insights into its potential antiviral therapeutic interventions.

## MATERIALS AND METHODS

### Cells and viruses

African green monkey renal cells (Vero E6), human embryonic kidney cells (HEK293T), and porcine intestinal epithelial cells (IPI-2I) were cultured in Dulbecco’s modified Eagle’s medium (11965092; Life Technologies, Carlsbad, CA, USA). RAW264.7 cells were cultured in Roswell Park Memorial Institute (RPMI) 1640 medium (11875093; Life Technologies). Human lung carcinoma cells (A549) were cultured in F-12K medium (21127022; Life Technologies). All media were supplemented with 10% fetal bovine serum (10099141C; Gibco, Life Technologies) at 37°C in a 5% CO_2_ environment. SADS-CoV (GenBank accession no. MF094681) was described previously (25), as were SeV and recombinant VSV-GFP (23).

### Antibodies and reagents

SADS-CoV N protein-specific monoclonal antibody 3E9 was prepared and maintained in our laboratory (26). Antibodies against Flag (ab205606), hemagglutinin (HA, ab9110), phosphorylated (p)-IRF3 (ab76493), IRF3 (ab68481), TBK1 (ab40676), p-TBK1 (ab109272), and GFP (ab290) were purchased from Abcam (Cambridge, MA, USA). Antibodies against Myc (M4439), HA (H9658), Flag (F1804), and glyceraldehyde 3-phosphate dehydrogenase (GAPDH, G9545) were purchased from Sigma-Aldrich (St. Louis, MO, USA). Antibodies against DDX11 (sc-271711) and COX IV (sc-376731) were purchased from Santa Cruz Biotechnology (Shanghai, China). Antibodies against MAVS (3993) and RIG-I (3743) were purchased from Cell Signaling Technology (Danvers, MA, USA). IRDye 800CW goat anti-mouse IgG secondary antibody (926-32210) and IRDye 680RD goat anti-rabbit IgG secondary antibody (925-68071) were purchased from LiCor BioSciences (Lincoln, NE, USA). RNase A, Alexa Fluor 594 goat anti-mouse IgG (H + L), Alexa Fluor 488 goat anti-rabbit IgG (H + L), and NeutrAvidin beads were purchased from Thermo Fisher Scientific (Waltham, MA, USA). Poly(I:C) and biotin-labeled poly(I:C) were purchased from InvivoGen (Hong Kong, China). Recombinant human IFN-β was purchased from R&D Systems (Minneapolis, MN, USA).

### Plasmid construction and transfection

Human full-length DDX11 (GenBank accession number: NM_001413699.1) and swine full-length DDX11 (GenBank accession number: XM_021092476.1) were amplified from the cDNA of HEK293T and IPI-2I cells, respectively. Subsequently, the DNA fragment of DDX11 was inserted into the pCAGGS-HA (P0166, Miaoling Biology) or pCMV-Myc (635689, Clontech) vector via homologous recombination using the ClonExpress ultra one-step cloning kit (C116; Vazyme, Nanjing, China). DDX11 truncation mutants were cloned on the basis of the full-length DDX11 plasmid and inserted into the pCAGGS-HA vector using the primers listed in Table S1. The genes encoding different swine DExD/H-box helicases were amplified from the cDNA of IPI-2I cells and inserted into the pCAGGS-HA vector. The IFN-β promoter luciferase reporter plasmid (IFN-β-Luc) and internal reference plasmid (pRL-TK) were stored in our laboratory as previously described (27). The plasmids for the expression of Flag-RIG-I(1-925aa), Flag-RIG-IN(1-283aa), Flag-RIG-IC(217-925aa), Flag-MDA5, Flag-IRF3, and Flag-TBK1 were preserved at our laboratory as previously described (23). MAVS truncation mutants were generated as previously described (28). The pcDNA3-tRNA scaffolded SA (tRSA) plasmid (32200) was purchased from Addgene (Watertown, MA, USA). SADS-CoV 3′-UTR and 5′-UTR were inserted into the pcDNA3-tRSA vector to generate the recombinant plasmids pcDNA3-tRSA-3′-UTR and pcDNA3-tRSA-5′-UTR, respectively. The accuracy of all plasmid constructs was verified by nucleotide sequencing analysis. HEK293T cells were transfected with the above plasmids using X-treme GENE HP (06366546001; Roche, Basel, Switzerland), while IPI-2I cells were transfected by Lipofectamine 3000 transfection reagent (L3000001; Thermo Fisher Scientific).

### RNA interference

Three small interfering RNAs (siRNAs) targeting DDX11 were designed by RiboBio (Guangdong, China). The siRNA target sequences used in the experiments are listed in Table S2. Cells were cultured in 12-well plates until growth was 40%–50% confluent and then transfected with siRNAs using the Lipofectamine RNAiMAX transfection reagent (13778075; Thermo Fisher Scientific) for 36 h. Cells were infected with SADS-CoV at the indicated multiplicity of infection for 24 h. Western blotting and quantitative real-time reverse transcription PCR (qRT-PCR) were performed to determine the effect of knockdown on DDX11.

### Generation of DDX11^−/−^, RIG-I^−/−^, and MAVS^−/−^ HEK293T cells by CRISPR/Cas9

To generate DDX11^−/−^, RIG-I^−/−^, and MAVS^−/−^ HEK293T cells, the CRISPR/Cas9 gene-editing system was used. The single-guide RNA (sgRNA) sequences used in the experiments are listed in Table S3. The sgRNA was cloned into the LentiCRISPRV2 (98290, Addgene) vector. HEK293T cells were co-transfected with 8 µg LentiCRISPRV2-sgRNA plasmid, 6 µg psPAX2 (12260, Addgene) plasmid, and 4 µg pVSV-G (138479, Addgene) plasmid. The produced lentivirus was collected 48 h post-transfection and concentrated with the Lenti-X Concentrator (631231; TaKaRa Bio, Shiga, Japan). HEK293T cells were then infected with lentivirus. At 2 days post-infection, the cells were selected using 4 µg/mL puromycin dihydrochloride (ST551; Beyotime Biotechnology, Shanghai, China). After pressurized screening, positive (puromycin-resistant) cells were individually sorted into single clones on a 96-well plate by flow cytometry using the model SH800S flow cell sorter (Sony, Tokyo, Japan). The knockout cells were validated by PCR and subsequently evaluated for protein expression by western blotting.

### Cell viability assays

The cell viability was determined using the CellTiter-Glo luminescent cell viability assay kit (G7570; Promega, Madison, WI, USA) according to the manufacturer’s instructions. Briefly, WT or DDX11^−/−^ HEK293T cells were grown in 96-well plates and then allowed to adhere to the plates. Cells were harvested at different time points and lysed using 100 µL of CellTiter-Glo reagent for 10 min at room temperature. Afterward, the luminescence signal was recorded using Multimode microplate reader (Envision, USA).

### Immunofluorescence assay and confocal microscopy

WT or MAVS^−/−^ HEK293T cells were grown in 35-mm diameter dishes that were pretreated with polylysine. The cells were transfected with the indicated plasmids or infected with SeV for 12 h. For mitochondrial staining, live cells were incubated with 150 mM MitoTracker Red CMXRos (M7512; Invitrogen, Carlsbad, CA, USA) at 37°C for 45 min. Afterward, cells were fixed with 4% paraformaldehyde (16005; Sigma-Aldrich) for 30 min at 4°C and then permeabilized with 0.1% Triton X-100 at room temperature for 15 min. Cells were blocked with 5% bovine serum albumin (ST025; Beyotime Biotechnology) for 1 h and incubated with special primary antibodies at 4°C overnight, followed by incubation with the corresponding fluorescein-conjugated secondary antibodies for 45 min at 37°C. Finally, nuclear DNA was stained with 4′,6-diamidino-2-phenylindole for 15 min at 37°C, and the cells were observed with an LSM880-ZEISS confocal laser scanning microscope (Carl Zeiss AG, Oberkochen, Germany).

### Western blotting and Co-IP

Whole cell lysates were lysed at indicated times after infection or transfection using radioimmunoprecipitation assay buffer (R0278; Sigma-Aldrich) supplemented with 1 mM phenylmethylsulfonyl fluoride solution (ST506-2; Beyotime Biotechnology) for 20 min on ice. After centrifugation for 15 min at 12,000 × *g*, supernatants were collected and boiled with 5× sodium dodecyl sulfate-polyacrylamide gel electrophoresis (SDS-PAGE) sample loading buffer (P0015L; Beyotime Biotechnology) for 10 min. Equal amounts of total proteins were resolved by SDS-PAGE and transferred to nitrocellulose filter membranes (66485; Pall Corporation, Port Washington, NY, USA) in transfer buffer at 250 mA for 100 min. The membranes were blocked with 5% skim milk (P0216; Beyotime Biotechnology) for 2 h at room temperature and incubated with primary antibodies at 4°C overnight. The membranes were washed five times and incubated with IRDye 800CW goat anti-mouse IgG secondary antibody (1:10,000) or IRDye 680RD goat anti-rabbit IgG secondary antibody (1:10,000) at room temperature for 1 h. The immunolabeled proteins were visualized using an Odyssey infrared imaging system (LiCor BioSciences, USA). For co-immunoprecipitation (Co-IP) assays, HEK293T cells transfected with indicated plasmids were lysed in IP lysis buffer (87788; Thermo Fisher Scientific) containing 1 mg/mL protease inhibitor cocktail (04693132001; Roche) for 30 min on ice. After centrifugation at 12,000 × *g* for 10 min at 4°C, the supernatant was collected and incubated with the indicated primary antibody with rotation at 4°C overnight. Subsequently, lysates were incubated with protein A/G magnetic beads (78609; Thermo Fisher Scientific) for 6 h. The beads were washed five times with cell lysis buffer (P0013; Beyotime Biotechnology). Immunoprecipitates were added to appropriately 1× SDS-PAGE sample loading buffer and boiled for 10 min for western blotting analysis.

### RNA extraction, reverse transcription, and qRT-PCR

Total RNA was extracted from cells employing a Simply P total RNA extraction kit (BSC52S1; BioFlux, Brussels, Belgium). One microgram of RNA was used for reverse transcription with the PrimeScript IV 1st strand cDNA Synthesis Mix (6215A; TaKaRa Bio). The qRT-PCR was performed on a QuantStudio 5 system (Applied Biosystems, Foster City, CA, USA) instrument with TB Green Premix Ex Taq II (RR820A; TaKaRa Bio). The relative quantification of gene expression was assessed using the comparative cycle threshold (ΔΔC_T_) method, with GAPDH serving as the endogenous reference gene for normalization. The specific primer sequences for the targeting genes are listed in Table S4.

### IFN-β ELISA

WT, DDX11^−/−^, and MAVS^−/−^ HEK293T cell lines were infected with SeV, and cell supernatants were collected at different time points post-infection. The secretion of IFN-β in the supernatant was detected with the human IFN-β ELISA kit (DIFNB0; R&D Systems, Minneapolis, MN, USA).

### IFN-β luciferase reporter assay

HEK293T cells were seeded into 24-well plates and transfected with 50 ng of luciferase reporter plasmid IFN-β-Luc and 5 ng of internal reference plasmid pRL-TK, together with the indicated plasmid alone or combined with the HA-DDX11 expression plasmid for 24 h. The cells were lysed for the assessment of luciferase activity with a dual luciferase reporter assay kit (E1901; Promega, Madison, WI, USA).

### Flow cytometry

HEK293T cells were transfected with the indicated plasmids and then infected with VSV-GFP for 12 h. Subsequently, the cells were digested with trypsin and resuspended in PBS. Cellular samples were gated to detect GFP signals, utilizing the background signal from non-infected cells as a reference. Fluorescence intensity was quantified using a Cytomics FC 500 flow cytometer (Beckman Coulter Inc., Brea, CA, USA). The data were analyzed using FlowJo software (FlowJo, Eugene, OR, USA).

### Cytoplasmic and mitochondrial isolation assay

WT or MAVS^−/−^ HEK293T cells were cultured in 6-well plates for 36 h and were mock infected or infected with SeV for 12 h. The cytosolic and mitochondrial fractions were isolated using a Mitochondria Isolation Kit (89874; Thermo Fisher Scientific). The specific operational steps have been previously detailed (25).

### Poly(I:C) pull-down assay

HEK293T cells were transfected with full-length or truncated DDX11 expression plasmids. Whole cell lysates were incubated with biotin-labeled poly(I:C) on a roller at 4°C for 4 h. NeutrAvidin beads were added for an additional 4 h at 4°C. After incubation, the beads were washed five times with lysis buffer and boiled in 1× SDS-PAGE sample loading buffer for western blotting analysis.

### RNA binding protein immunoprecipitation assay

HEK293T cells were transfected with the HA-DDX11 or Flag-RIG-I expression plasmid for 24 h, followed by VSV-GFP or SADS-CoV infection for 12 or 24 h, respectively. Cells were harvested in IP lysis buffer containing 1 mM polymethyl sulfonyl fluoride and an RNase inhibitor (R0102; Beyotime Biotechnology). Co-IP was performed as described earlier, and 10% of the whole cell lysate was collected for RNA and protein inputs. The remaining cell lysate was combined with beads and antibodies to form a beads-antibody-cell lysate mixture, and then the beads were washed six times with IP lysis buffer. The collected beads were resuspended and divided into two samples for either protein or RNA extraction. Proteins were extracted for western blotting analysis, while the RNA extraction was performed using TRIzol RNA extraction reagent (10296028CN; Thermo Fisher Scientific).

### RNA pull-down

The 5′-UTR and 3′-UTR transcripts of SADS-CoV were synthesized *in vitro*. Briefly, the cDNA corresponding to 5′ UTR and 3′ UTR of SADS-CoV was inserted into the pcDNA3-tRSA vector. These constructs were amplified via PCR to generate linear templates, which were utilized for *in vitro* synthesis of 5′-UTR and 3′-UTR transcripts using a HyperScribe T7 High Yield RNA Synthesis Kit (K1047; APExBIO, Houston, TX, USA). The purified RNA was used with the Pierce RNA 3′ End Desthiobiotinylation Kit (20163; Thermo Fisher Scientific) to attach a single biotinylated nucleotide to the 3′ terminus of an RNA strand for RNA labeling. Afterward, the labeled RNA was captured with streptavidin magnetic beads using a Pierce magnetic RNA-protein pull-down kit (20164; Thermo Fisher Scientific). The labeled RNA-bound beads were equilibrated in protein-RNA binding buffer, mixed with an equal volume of cell lysates from HEK293T cells transfected with HA-DDX11, and treated with lysis buffer. The biotinylated RNA/protein complexes were incubated on a roller for 2 h at 4°C, followed by three washes with 1× washing buffer. The RNA binding protein was analyzed by western blotting.

### Viral titration

The supernatant of HEK293T or IPI-2I cells infected with SADS-CoV was collected for virus titration. Briefly, Vero E6 cells were cultured in 96-well plates to 80% confluency and washed three times with PBS. Then, the cells were infected with 10-fold serial dilutions of the supernatants, using eight replicates per dilution. At 4–6 days post-infection, the viral cytopathic effect was observed by light microscopy, and the TCID_50_/mL was calculated using the Reed and Muench method.

### Statistical analysis

The results of three independent experiments were analyzed for experimental data, calculation of mean ± standard deviation (SD), and graph plotting using GraphPad Prism software (version 9.0; GraphPad, San Diego, CA, USA). Error bars represent SD. According to the experiment, *P*-values are *P < 0.05; **P < 0.01; ***P < 0.001; ****P < 0.0001; ns, not significant.

## RESULTS

### Role of DExD/H-box helicases during RNA virus infection

**Fig 1:**
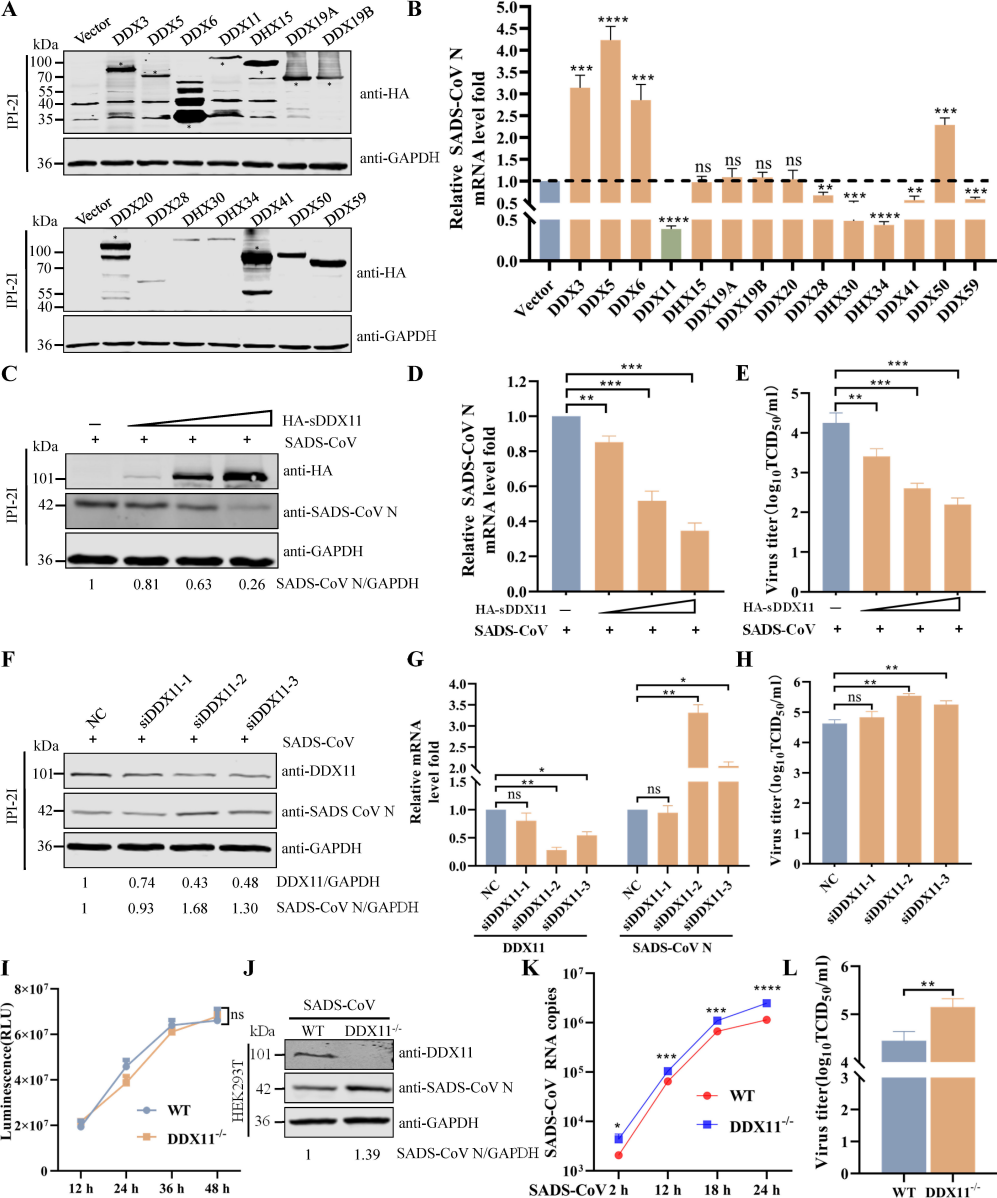
Screening for DExD/H-box helicases that regulate SADS-CoV infection. (**A and B**) IPI-2I cells were transfected with eukaryotic expression plasmids encoding various DExD/H-box helicases or empty vector for 24 h and then infected with SADS-CoV at a multiplicity of infection (MOI) of 0.1 for 24 h. (**A**) Protein expression of different DExD/H-box helicases detected by western blotting. (**B**) The mRNA levels of SADS-CoV N protein verified by qRT-PCR. (**C–E**) Overexpression of DDX11 inhibits SADS-CoV replication. IPI-2I cells were transfected with HA-tagged swine DDX11 at concentrations of 0.5, 1, and 2 µg or with a control empty vector for 24 h and then infected with SADS-CoV at an MOI of 0.1 for 24 h. (**C**) Protein expression levels of both DDX11 and SADS-CoV N analyzed by western blotting. Calculated band density values for SADS-CoV N/GAPDH; the values of the empty vector-transfected group are standardized to one. (**D**) The mRNA levels of the SADS-CoV N protein validated by qRT-PCR. (**E**) The SADS-CoV TCID_50_ in the supernatants was titrated on Vero E6 cells. (**F–H**) Knockdown of DDX11 enhances SADS-CoV replication. IPI-2I cells were transfected with siDDX11-1, siDDX11-2, siDDX11-3, or siNC (negative control) at 50 nM for 36 h and then infected with SADS-CoV at an MOI of 0.1 for 24 h. (**F**) Protein expression levels of both DDX11 and SADS-CoV N analyzed by western blotting. Calculated band density values for DDX11/GAPDH and SADS-CoV N/GAPDH; the values of the siNC group are standardized to one. (**G**) The mRNA levels of DDX11 and SADS-CoV N detected by qRT-PCR. (**H**) The SADS-CoV TCID_50_ in the supernatants was titrated on Vero E6 cells. (**I–K**) Knockout of DDX11 enhances SADS-CoV replication. WT HEK293T and DDX11^−/−^ HEK293T cells were infected with SADS-CoV at an MOI of 1 for 24 h. (**I**) Cellular proliferation was detected in WT or DDX11^−/−^ HEK293T cells, respectively, using CellTiter-Glo at the indicated time points. (**J**) Protein expression levels of both DDX11 and SADS-CoV N determined by western blotting. Calculated band density values for SADS-CoV N/GAPDH; the values of WT HEK293T cells group are standardized to one. (**K**) The viral RNA copy number was tested by qRT-PCR at 2, 12, 18, and 24 h post-infection. (**L**) The SADS-CoV TCID_50_ in the supernatants was titrated on Vero E6 cells. Means and SD (error bars) of three independent experiments are indicated (*P < 0.05; **P < 0.01; ***P < 0.001; ****P < 0.0001; ns, not significant).

DExD/H-box helicases play a critical role in antiviral innate immune responses (29). Several studies described the involvement of DExD/H-box helicases family members in the replication process of different viruses (30–32). To comprehensively gauge the potential impacts of various DExD/H-box helicases on virus infection, IPI-2I cells were transfected with expression plasmids encoding one each of 14 different swine DExD/H-box helicases and subsequently infected with SADS-CoV. Western blotting showed that the individual DExD/H-box helicase proteins were successfully expressed (Fig. 1A). Quantitative real-time reverse transcription PCR results indicated that ectopic expression of DDX11 and DHX34 remarkably inhibited the mRNA levels of SADS-CoV N protein compared with empty vector, while DDX3, DDX5, DDX6, and DDX50 substantially promoted the mRNA levels of SADS-CoV N protein (Fig. 1B). To assess the biological relevance of DDX11 in viral infection, we analyzed the effects of its overexpression on the replication of SADS-CoV. Western blotting showed that the level of viral N protein in SADS-CoV-infected IPI-2I cells was significantly decreased in a dose-dependent manner by DDX11 overexpression (Fig. 1C). Consistently, qRT-PCR and 50% tissue infective dose (TCID_50_) assays also showed that DDX11 overexpression decreased the level of SADS-CoV N mRNA and viral titer of SADS-CoV (Fig. 1D and E). To further investigate the antiviral role of DDX11 on SADS-CoV replication, we designed three small interfering RNAs targeting swine DDX11 to downregulate DDX11 expression in IPI-2I cells. Both siDDX11-2 and siDDX11-3 significantly decreased DDX11 protein and mRNA levels in transfected IPI-2I cells (Fig. 1F and G). Knockdown of DDX11 led to a significant increase in the expression of SADS-CoV N protein (Fig. 1F and G) and enhanced the release of infectious virus (Fig. 1H). Moreover, cells of an independently generated DDX11 knockout cell line (DDX11^−/−^ HEK293T cells) showed no proliferation defects (Fig. 1I). DDX11^−/−^ HEK293T cells exhibited elevated levels of viral protein and mRNA, along with increased viral yield, in comparison to wild-type (WT) HEK293T cells (Fig. 1J through L). Collectively, these results indicated the significant antiviral role of DDX11 on the RNA virus.

### DDX11 positively regulates SeV and poly(I:C)-induced IFN response

**Fig 2:**
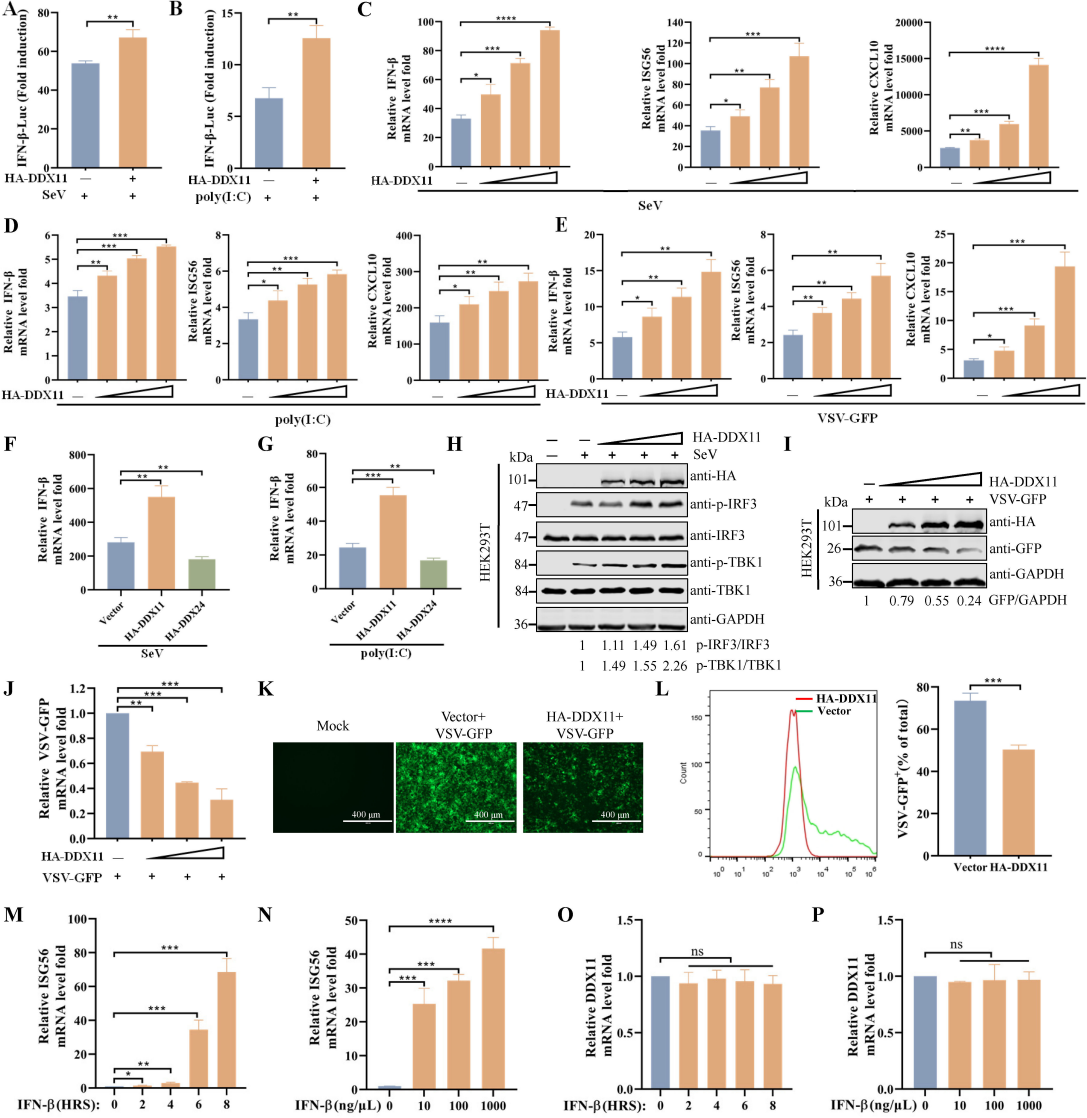
DDX11 positively regulates SeV and poly(I:C)-induced IFN response. (**A and B**) Overexpression of DDX11 potentiates the activity of the IFN-β promoter in response to stimulation by SeV and poly(I:C). HEK293T cells were transfected with empty vector or HA-DDX11, along with IFN-β-Luc and pRL-TK for 24 h. Cells were infected with SeV (**A**) or stimulated with poly(I:C) (**B**) for 12 h. Luciferase activity was quantified utilizing a dual luciferase reporter assay system. (**C–E**) Overexpression of DDX11 increases the transcription of IFN-β, ISG56, and CXCL10. HEK293T cells were transfected with HA-DDX11 for 24 h and then stimulated with SeV (**C**), poly(I:C) (**D**), or VSV-GFP (**E**) for 12 h. The transcription levels of IFN-β, ISG56, and CXCL10 were evaluated by qRT-PCR. (**F and G**) Overexpression of DDX24 inhibits the mRNA expression levels of IFN-β. HEK293T were transfected with empty vector, HA-DDX11, or HA-DDX24 for 24 h and then infected with SeV (**F**) or stimulated with poly(I:C) (**G**) for 12 h. The cells were harvested for qRT-PCR to detect the mRNA levels of IFN-β. (**H**) Overexpression of DDX11 increases the phosphorylation of IRF3 and TBK1 stimulated by SeV. HEK293T cells were transfected with empty vector or HA-DDX11 for 24 h and then stimulated with SeV for 12 h. The cell lysates were analyzed by western blotting. The band density values for p-IRF3/IRF3 and p-TBK1/TBK1 were calculated, with the values of the empty vector-treated group being standardized to one. (**I–L**) Overexpression of DDX11 inhibits VSV-GFP replication. HEK293T were transfected with empty vector or HA-DDX11 for 24 h and then infected with VSV-GFP for 12 h. (**I**) Protein expression levels of both DDX11 and GFP were detected by western blotting. The band density values for GFP/GAPDH were calculated, with the values of the empty vector-treated group being standardized to one. (**J**) The mRNA levels of VSV-GFP were verified by qRT-PCR. (**K**) The propagation of VSV-GFP was monitored and characterized by fluorescence microscopy (bar: 400 µm). (**L**) Fluorescence was analyzed by flow cytometry. (**M–P**) Exogenous addition of recombinant IFN-β increases mRNA levels of ISG56, but not DDX11. HEK293T cells were treated with recombinant IFN-β (100 ng/µL) for 0, 2, 4, 6, and 8 h. The mRNA levels of ISG56 (**M**) and DDX11 (**O**) were analyzed by qRT-PCR. HEK293T cells were treated with recombinant IFN-β (0, 10, 100, and 1,000 ng/µL) for 8 h, and the mRNA levels of ISG56 (**N**) and DDX11 (**P**) were analyzed by qRT-PCR. Means and SD (error bars) of three independent experiments are indicated (*P < 0.05; **P < 0.01; ***P < 0.001; ****P < 0.0001; ns, not significant).

To directly investigate the underlying anti-SADS-CoV mechanism of DDX11, DDX11 was overexpressed in HEK293T cells. As shown in Fig. 2A and B, this overexpression potentiated SeV- or poly(I:C)-induced activation of the IFN-β promoter. The qRT-PCR analysis indicated that overexpression of DDX11 potentiated SeV-, poly(I:C)-, or VSV-GFP-triggered the transcription of downstream genes, including IFN-β, ISG56, and CXCL10 in HEK293T cells (Fig. 2C through E). Conversely, overexpression of DDX24 decreased SeV- or poly I:C-induced transcription of IFN-β (Fig. 2F and G), as DDX24 has been identified as a negative regulator for RLR-mediated innate immune signaling (14). Consistent with these results, the ectopic expression of DDX11 significantly enhanced IRF3 and TBK1 phosphorylation in response to SeV infection (Fig. 2H). Additionally, HEK293T cells that ectopically expressed DDX11 infected with VSV-expressing green fluorescent protein (VSV-GFP) were constructed. Western blotting showed that overexpression of DDX11 significantly decreased the protein levels of VSV-GFP in a dose-dependent manner (Fig. 2I). qRT-PCR, fluorescence microscopy, and flow cytometry showed that overexpression of DDX11 substantially inhibited the replication of VSV-GFP compared to vector-transfected cells (Fig. 2J through L). Given the inherent susceptibility of VSV-GFP to the antiviral effect of IFN, and considering that DDX11 restricted VSV-GFP replication, it was hypothesized that the expression of DDX11 was regulated by IFN. The hypothesis was assessed by analyzing the expression of DDX11 in IFN-β-treated HEK293T cells by qRT-PCR. As shown in Fig. 2M through P, IFN-β treatment led to increased ISG56 transcript levels in time- and dose-dependent manners, but not DDX11, indicating DDX11 was not an ISG under these conditions. Collectively, these results suggested that DDX11 positively regulated SeV- and poly(I:C)-induced IFN responses and had antiviral action.

### DDX11 knockdown or knockout reduces SeV and poly(I:C)-induced IFN response in different cells

**Fig 3:**
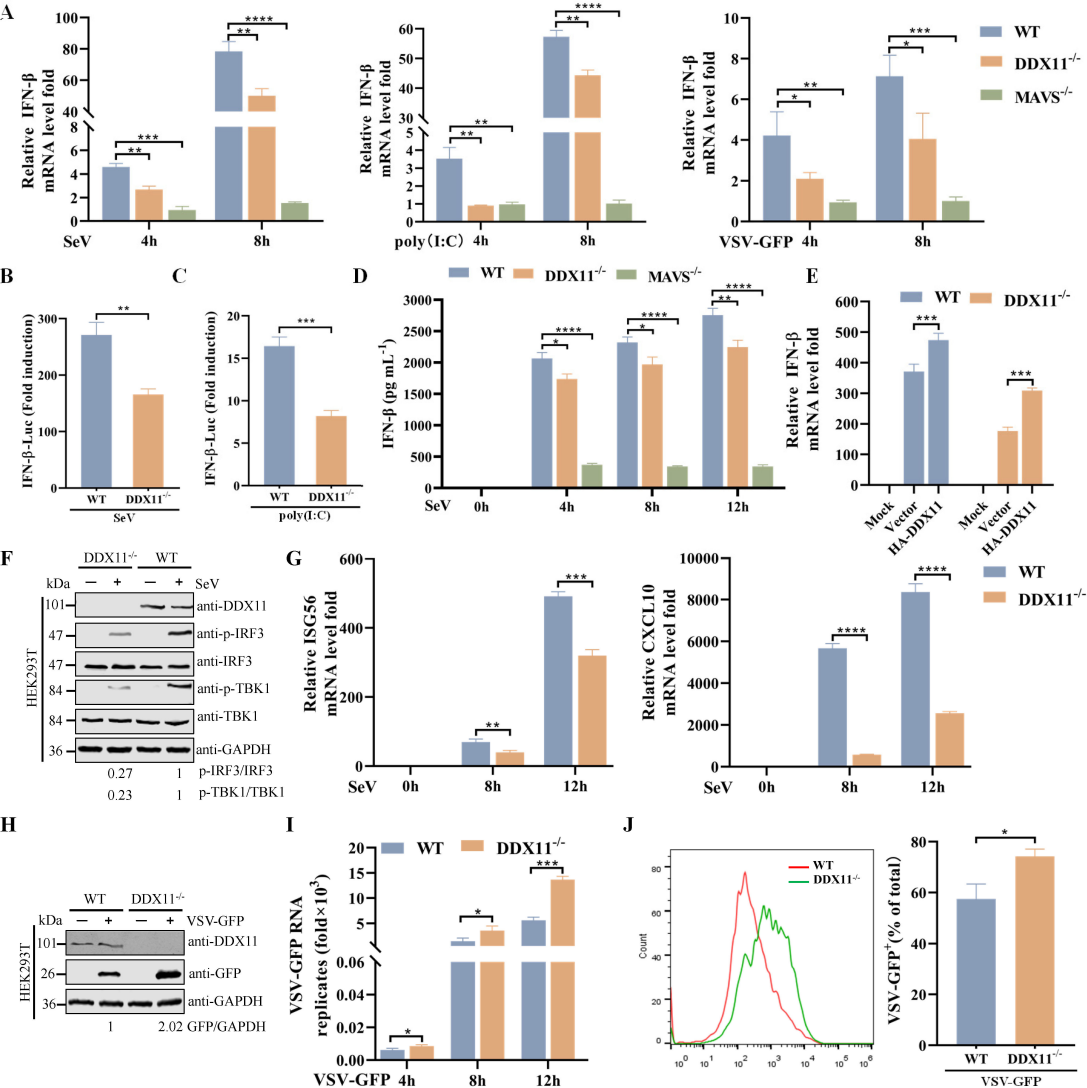
DDX11 knockout reduces SeV- and poly(I:C)-induced IFN response. (**A**) Knockout of DDX11 and MAVS reduces the transcription of IFN-β after SeV or VSV-GFP infection and poly(I:C) transfection. WT HEK293T, DDX11^−/−^ HEK293T, and MAVS^−/−^ HEK293T cells were infected with SeV and VSV-GFP or stimulated with poly(I:C) for 4 and 8 h. The mRNA levels of IFN-β were analyzed by qRT-PCR. (**B and C**) Knockout of DDX11 diminishes the activity of the IFN-β promoter in response to SeV infection (**B**) and poly(I:C) (**C**) stimulation in HEK293T cells. Luciferase activity was quantified utilizing a dual luciferase reporter assay system. (**D**) Knockout of DDX11 and MAVS reduces IFN-β secretion after SeV infection. The IFN-β protein in the supernatants of SeV-infected WT HEK293T, DDX11^−/−^ HEK293T, and MAVS^−/−^ HEK293T cells was detected with the human IFN-β quantikine ELISA kit. (**E**) Overexpression of DDX11 in DDX11^−/−^ HEK293T cells restores the inhibitory effect of knockout of DDX11 on IFN-β. WT HEK293T and DDX11^−/−^ HEK293T cells were transfected with an empty vector or HA-DDX11 and then infected with SeV for 12 h. The mRNA levels of IFN-β were analyzed by qRT-PCR. (**F**) Knockout of DDX11 reduces phosphorylation of IRF3 and TBK1 stimulated by SeV. WT HEK293T and DDX11^−/−^ HEK293T cells were stimulated with SeV for 12 h and then analyzed by western blotting. The band density values for p-IRF3/IRF3 and p-TBK1/TBK1 were calculated, with the values of SeV-infected WT HEK293T cell group being standardized to one. (**G**) Knockout of DDX11 inhibits the transcription of ISG56 and CXCL10 after SeV infection. WT HEK293T and DDX11^−/−^ HEK293T cells were stimulated with SeV for 8 and 12 h, and the relative mRNA levels of ISG56 and CXCL10 were evaluated by qRT-PCR. (**H–J**) Knockout of DDX11 promotes VSV-GFP replication. WT HEK293T and DDX11^−/−^ HEK293T cells were infected with VSV-GFP. (**H**) Protein expression levels of DDX11 and GFP were detected by western blotting. The band density values for GFP/GAPDH were calculated, with the values of VSV-GFP-infected WT HEK293T cell group being standardized to one. (**I**) VSV-GFP RNA transcripts were analyzed by qRT-PCR. (**J**) Fluorescence was analyzed by flow cytometry. Means and SD (error bars) of three independent experiments are indicated (*P < 0.05; **P < 0.01; ***P < 0.001; ****P < 0.0001; ns, not significant).

Given that overexpression of DDX11 positively regulated the IFN response, it was interesting to investigate whether knockdown or knockout of DDX11 also affected this response. Transfection with DDX11-specific small interfering RNAs resulted in a significant reduction in both protein and mRNA levels of endogenous DDX11 in RAW264.7 and A549 cells, compared to cells transfected with negative control (siNC) (Fig. S1A through D). In contrast to the overexpression of DDX11, siRNA knockdown of DDX11 expression significantly decreased the level of IFN-β mRNA expression in RAW264.7 and A549 cells after SeV infection or poly(I:C) stimulation (Fig. S1E through H). DDX11^−/−^ and MAVS^−/−^ HEK293T cells were used to directly confirm that DDX11 was affecting IFN signaling. As shown in Fig. 3A, WT HEK293T cells induced higher levels of IFN-β mRNA than DDX11^−/−^ and MAVS^−/−^HEK293T cells in response to SeV and VSV-GFP infection or poly(I:C) stimulation. Furthermore, knockout of DDX11 significantly decreased the activation of IFN-β (Fig. 3B and C). Meanwhile, we found that the release of IFN-β was significantly reduced in the DDX11^−/−^ and MAVS^−/−^ HEK293T cells infected with SeV at different time points (Fig. 3D). Overexpression of DDX11 in DDX11^−/−^ HEK293T cells restored the inhibitory effect of knockdown of DDX11 on IFN-β transcript levels (Fig. 3E). Consistent with the above results, the phosphorylation of TBK1 and IRF3 induced by SeV in DDX11^−/−^ HEK293T cells was markedly decreased in comparison with WT HEK293T (Fig. 3F). In addition, knockout of DDX11 also markedly reduced ISG56 and CXCL10 transcription in response to SeV (Fig. 3G). Because knockdown or knockout of DDX11 suppressed the production of IFN-β induced by SeV and poly(I:C), the potential regulatory role of DDX11^−/−^ HEK293T cells on viral infection and proliferation was investigated. Western blotting, qRT-PCR, and flow cytometry showed that DDX11^−/−^ HEK293T cells facilitated VSV-GFP replication compared with WT HEK293T cells (Fig. 3H through J). The collective results of knockdown or knockout of DDX11 in different cells clearly suggested that DDX11 was required for IFN signaling.

### DDX11 targets RIG-I and MAVS

**Fig 4:**
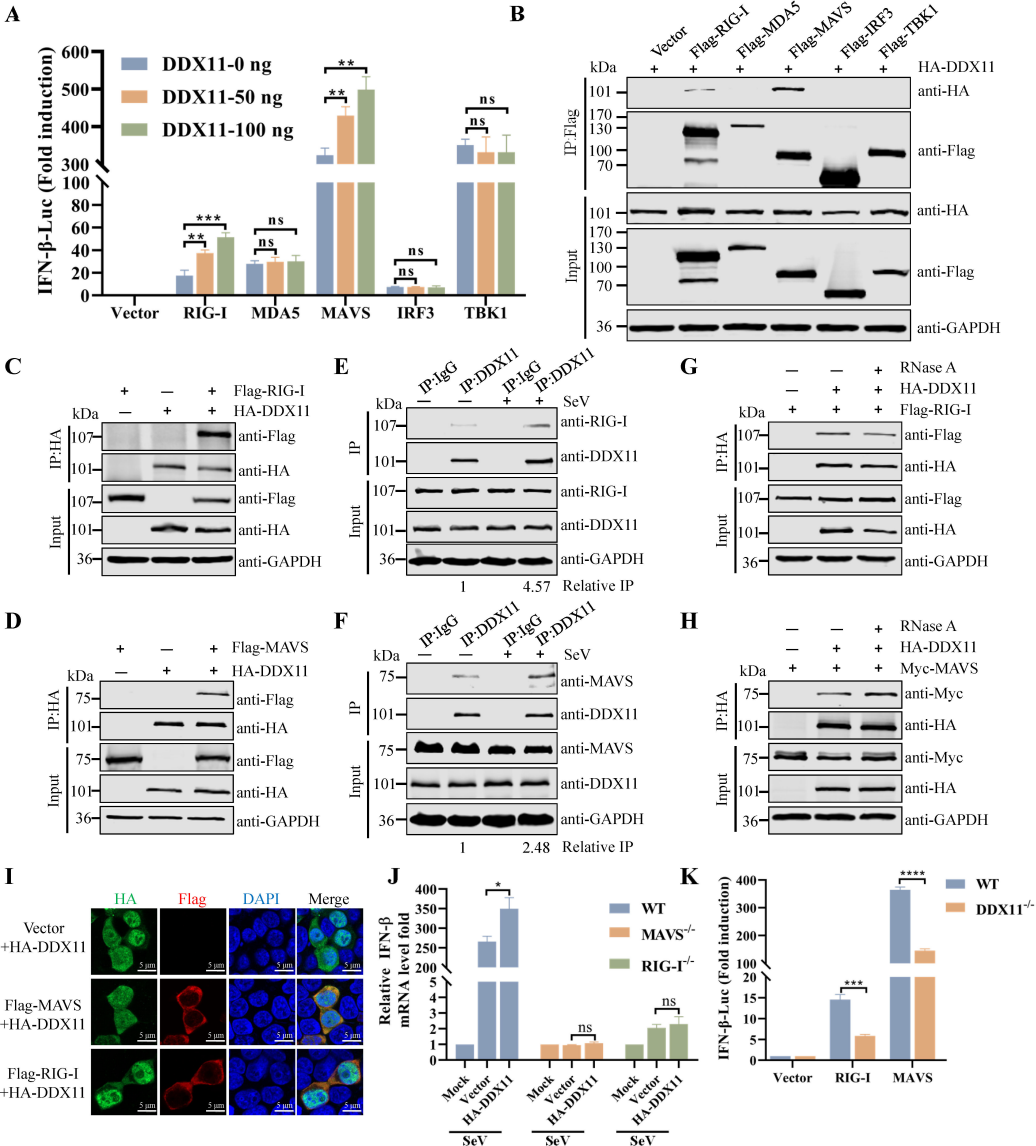
DDX11 targets RIG-I and MAVS. (**A**) Overexpression of DDX11 increases the IFN-β promoter activation mediated by RIG-I and MAVS. HEK293T cells were transfected with IFN-β-Luc (50 ng), pRL-TK (5 ng), and 100 ng of each plasmid expressing RIG-I, MDA5, MAVS, IRF3, or TBK1, along with an empty vector or a plasmid expressing DDX11 (0, 50, and 100 ng) for 24 h. Luciferase activity was quantified utilizing a dual luciferase reporter assay system. (**B**) Screening for key molecules in the RLRs signaling pathway that interact with DDX11. HEK293T cells were transfected with plasmids expressing RIG-I, MDA5, MAVS, IRF3, TBK1, or empty vector, together with HA-DDX11. Cellular extracts were harvested and subsequently subjected to immunoprecipitation using an anti-Flag antibody. The immunoprecipitants were analyzed by western blotting using relevant specific antibodies. (**C**) DDX11 interacts with RIG-I. HEK293T cells were co-transfected with Flag-RIG-I and HA-DDX11 for 24 h. Co-IP and immunoblot analyses of the interaction of DDX11 with RIG-I. (**D**) Co-IP and immunoblot analyses of the interaction of DDX11 with MAVS. (**E and F**) Endogenous DDX11 interacts with RIG-I and MAVS. HEK293T cells were infected with SeV for 12 h. The cells were harvested, and Co-IP was performed with anti-DDX11 antibody. IgG was used as a negative control. The immunoprecipitants were analyzed by western blotting using relevant specific antibodies. The band density values for interacting protein/IP protein in the IP samples are presented below the corresponding figure. (**G and H**) Co-IP and immunoblot analyses of the interaction of DDX11 with RIG-I (**G**) and MAVS (**H**) in the presence of RNase A (100 mg/mL). (**I**) Interaction of DDX11 with RIG-I and MAVS assessed by confocal microscopy (Bar: 5 µm). (**J**) WT HEK293T, MAVS^−/−^ HEK293T, and RIG-I^−/−^ HEK293T cells were transfected with HA-DDX11 and then infected with SeV for 12 h. The mRNA levels of IFN-β were analyzed by qRT-PCR. (**K**) WT HEK293T and DDX11^−/−^ HEK293T cells were transfected with plasmid expressing RIG-I and MAVS or empty vector, together with IFN-β-Luc and pRL-TK for 24 h. Luciferase activity was quantified utilizing a dual luciferase reporter assay system. Means and SD (error bars) of three independent experiments were indicated (*P < 0.05; **P < 0.01; ***P < 0.001; ****P < 0.0001; ns, not significant).

To elucidate the functional mechanisms of DDX11 in the innate antiviral response, the effects of DDX11 on IFN-β reporter activation induced by key molecules in the RLR signaling pathways were evaluated. As shown in Fig. 4A, ectopically expressed DDX11 significantly promoted IFN-β reporter activation induced by RIG-I and MAVS in a dose-dependent manner; the reporter activation was not induced by MDA5, IRF3, and TBK1. Consistent with these results, co-immunoprecipitation assays revealed the specific interaction of DDX11 with RIG-I and MAVS (Fig. 4B). To further test this possibility, the interaction between DDX11 and RIG-I or MAVS was assessed by Co-IP. DDX11 interacted with RIG-I and MAVS (Fig. 4C and D). Endogenous DDX11 interacted with low amounts of RIG-I and MAVS in uninfected cells. These interactions were enhanced after SeV infection (Fig. 4E and F). Since DDX11 and RIG-I have a strong affinity for nucleic acids, the DDX11-RIG-I and DDX11-MAVS interaction might have been mediated through the formation of a nonspecific RNA bridge. However, RNase A treatment of cell extracts before IP did not reduce the DDX11-RIG-I (Fig. 4G) or DDX11-MAVS (Fig. 4H) association, indicating that the interaction is not due to nonspecific RNA-mediated effects. Additionally, confocal microscopy revealed that DDX11 co-localized with RIG-I and MAVS (Fig. 4I). RIG-I^−/−^ and MAVS^−/−^ HEK293T cells were used to further determine if DDX11 targeted RIG-I and MAVS to induce IFN production. The qRT-PCR analyses indicated that overexpressed DDX11 could induce the mRNA expression of IFN-β in WT HEK293T cells, but not in RIG-I^−/−^ and MAVS^−/−^ HEK293T cells (Fig. 4J). In addition, the deficiency of DDX11 inhibited RIG-I- and MAVS-induced activation of the IFN-β promoter (Fig. 4K). These collective results suggested that DDX11 interacted with RIG-Ι and MAVS and that these associations were necessary for the functions of DDX11 in RIG-I-MAVS-mediated signaling.

### DDX11 acts as an RNA co-sensor to facilitate RIG-I recognition of viral RNA

**Fig 5:**
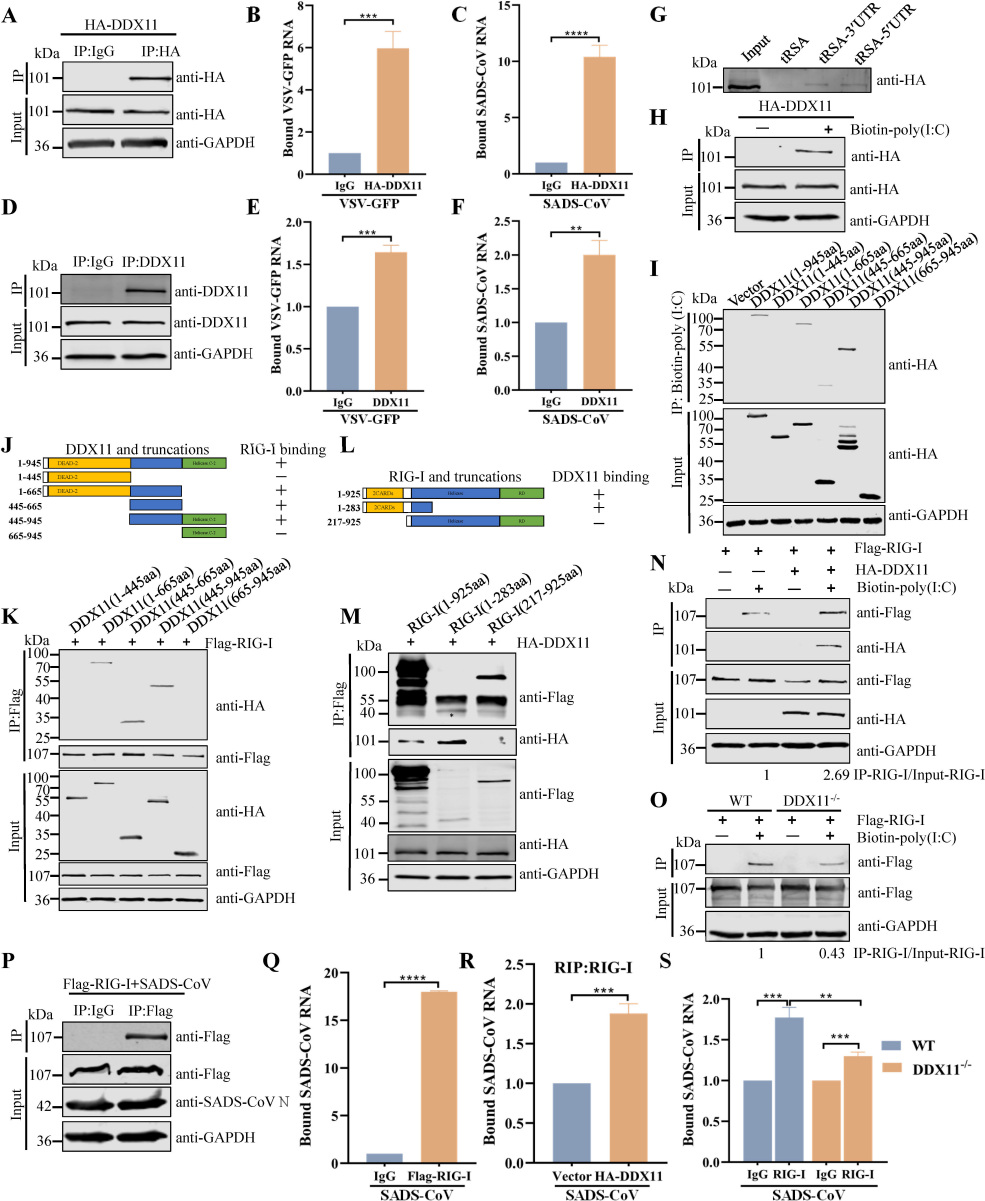
DDX11 acts as an RNA co-sensor to facilitate RIG-I recognition of viral RNA. (**A–C**) Exogenously expressed DDX11 binds to VSV-GFP-RNA and SADS-CoV-RNA. HEK293T cells were transfected with HA-DDX11 for 24 h and then infected with VSV-GFP for 12 h or SADS-CoV for 24 h. The cells were lysed, and immunoprecipitation was performed with anti-HA or anti-IgG antibody. (**A**) Detection of the immunoprecipitants by western blotting with the indicated antibodies. (**B and C**) The immunoprecipitants were analyzed by qRT-PCR to detect bound VSV-GFP-RNA (**B**) and SADS-CoV-RNA (**C**). (**D–F**) Endogenous DDX11 binds to VSV-GFP-RNA and SADS-CoV-RNA. HEK293T cells were infected with VSV-GFP for 12 h or SADS-CoV for 24 h. The cells were lysed, and immunoprecipitation was performed with anti-DDX11 or anti-IgG antibody. (**D**) The immunoprecipitants were detected by western blotting with indicated antibodies. (**E and F**) The immunoprecipitants were analyzed by qRT-PCR to detect bound VSV-GFP-RNA (**E**) and SADS-CoV-RNA (**F**). (**G**) DDX11 binds both the 5′ UTR and 3′ UTR transcripts of SADS-CoV. The 5′-UTR or 3′-UTR transcripts of SADS-CoV were labeled and then captured with streptavidin beads. The labeled RNA-bound beads were incubated with HEK293T cell lysates overexpressing HA-DDX11. The bound proteins were analyzed by western blotting with anti-HA antibody. (**H**) DDX11 binds to poly(I:C). HEK293T cells were transfected with HA-DDX11 for 24 h. The cell lysates were incubated with biotin-labeled poly(I:C) for 4 h at 4°C, followed by the addition of NeutrAvidin beads for 4 h. The proteins bound to the beads were analyzed by western blotting. (**I**) Amino acids 445–665 of DDX11 are required for the binding of DDX11 to poly(I:C). HEK293T cells were transfected with full-length HA-DDX11 or truncation of DDX11 for 24 h. Cell lysates were incubated with biotin-labeled poly(I:C) for 4 h at 4°C, followed by the addition of NeutrAvidin beads for 4 h. The proteins bound to beads were analyzed by western blotting. (**J**) Schematics of full-length DDX11 and its serial truncated mutants. (**K**) Identification of the domain of DDX11 required for the interaction with RIG-I. HEK293T cells were transfected with Flag-RIG-I and DDX11 truncations. Cell lysates were collected for Co-IP and immunoblot analyses. (**L**) Schematics of full-length RIG-I and its serial truncated mutants. (**M**) Identification of the domain of RIG-I required for the interaction with DDX11. (**N**) Overexpression of DDX11 increases RIG-I recognition of poly(I:C). HEK293T cells were transfected with Flag-RIG-I, together with HA-DDX11 or empty vector. Cell lysates were incubated with biotin-labeled poly(I:C) for 4 h. Immunoprecipitation was performed with NeutrAvidin beads, followed by immunoblotting with anti-HA or anti-Flag antibody. Relative quantification of the IP-RIG-I/Input-RIG-I is shown below. (**O**) Knockout of DDX11 decreases RIG-I recognition of poly(I:C). WT HEK293T cells and DDX11^−/−^ HEK293T cells were transfected with Flag-RIG-I for 24 h. Cell lysates were incubated with biotin-labeled poly(I:C) for 4 h. Immunoprecipitation was performed with NeutrAvidin beads, followed by immunoblotting with anti-Flag antibody. Relative quantification of the IP-RIG-I/Input-RIG-I is shown below. (**P and Q**) RIG-I binds to SADS-CoV-RNA. HEK293T cells were transfected with Flag-RIG-I for 24 h and then infected with SADS-CoV for 24 h. The cells were lysed, and immunoprecipitation was performed with an anti-Flag or anti-IgG antibody. (**P**) Detection of the immunoprecipitants by western blotting with the indicated antibodies. (**Q**) The immunoprecipitants were analyzed by qRT-PCR to detect bound SADS-CoV-RNA. (**R**) Overexpression of DDX11 increases RIG-I recognition of SADS-CoV-RNA. HEK293T cells were transfected with HA-DDX11 or empty vector for 24 h and then infected with SADS-CoV for 24 h. Immunoprecipitation was performed with anti-RIG-I antibody. The immunoprecipitants were analyzed by qRT-PCR to detect bound SADS-CoV-RNA. (**S**) Knockout of DDX11 decreases RIG-I recognition of SADS-CoV-RNA. WT HEK293T and DDX11^−/−^ HEK293T cells were infected with SADS-CoV for 24 h. The cells were lysed, and immunoprecipitation was performed with anti-RIG-I or anti-IgG antibody. The immunoprecipitants were analyzed by qRT-PCR to detect bound SADS-CoV-RNA. The mean and SD (error bars) of three independent experiments are indicated (*P < 0.05; **P < 0.01; ***P < 0.001; ****P < 0.0001; ns, not significant).

Given the identification of numerous DExD/H-box helicases as RNA virus sensors, we postulated that DDX11 may act as an RNA sensor through the direct recognition of viral RNA. To examine this, HEK293T cells were transfected with HA-DDX11 for 24 h, followed by infection with VSV-GFP or SADS-CoV. The cells were lysed, and immunoprecipitation was performed using anti-HA or anti-IgG antibodies (Fig. 5A). RNA levels of the bound viruses were subsequently measured by qRT-PCR, showing that DDX11 binds to the RNA of both VSV-GFP (Fig. 5B) and SADS-CoV (Fig. 5C). Furthermore, the binding experiments demonstrating the interaction of endogenous DDX11 with VSV-GFP-RNA and SADS-CoV-RNA provided additional support for our hypothesis (Fig. 5D through F). We then tested the interaction between DDX11 and the 5′ or 3′ untranslated region (UTR) transcripts of SADS-CoV. As shown in Fig. 5G, DDX11 could bind both the 5′-UTR or 3′-UTR transcripts of SADS-CoV. In addition, pull-down assays indicated that ectopically expressed DDX11 could bind to poly(I:C) (Fig. 5H). We also prepared truncated versions of DDX11 involving amino acids (aa) 1–445, 1–665, 445–665, 445–945, and 665–945. Deletion analysis indicated that the binding to poly(I:C) required the 445–665 aa domain of DDX11 (Fig. 5I). RIG-I comprises N-terminal two caspase activation and recruitment domains (2CARDs), a central DECH box ATPase domain, and a C-terminal regulatory/repressor domain. Domain mapping experiments indicated that the 2CARDs of RIG-I and the aa 445–665 domain of DDX11 were required for their interaction (Fig. 5J through M).

In order to elucidate the molecular mechanisms by which DDX11 promoted IFN signaling mediated by RIG-I, we incubated biotin-labeled poly(I:C) with cell lysate of HEK293T co-transfected with RIG-I along with DDX11 or empty vector and purified the preparation with avidin-conjugated beads. We found that HA-DDX11 and Flag-RIG-I alone or in combination could be immunoprecipitated with avidin-conjugated beads (Fig. 5N). Furthermore, RIG-I exhibited a strong poly(I:C)-binding ability upon overexpression of DDX11 (Fig. 5N). This strong binding was attenuated upon knockout of DDX11 (Fig. 5O). Pull-down and qRT-PCR assays showed that RIG-I served as a pattern recognition receptor to identify SADS-CoV-RNA (Fig. 5P and Q). In addition, overexpression of DDX11 promoted RIG-I recognition of SADS-CoV-RNA (Fig. 5R). Consistent with these results, knockout of DDX11 decreased the RIG-I-SADS-CoV-RNA interaction in response to SADS-CoV infection (Fig. 5S). Taken together, these results indicated that aa 445–665 of DDX11 were required for its function as an RNA co-sense to promote RIG-I recognition viral RNA.

### DDX11 promotes RIG-I and MAVS interaction

**Fig 6:**
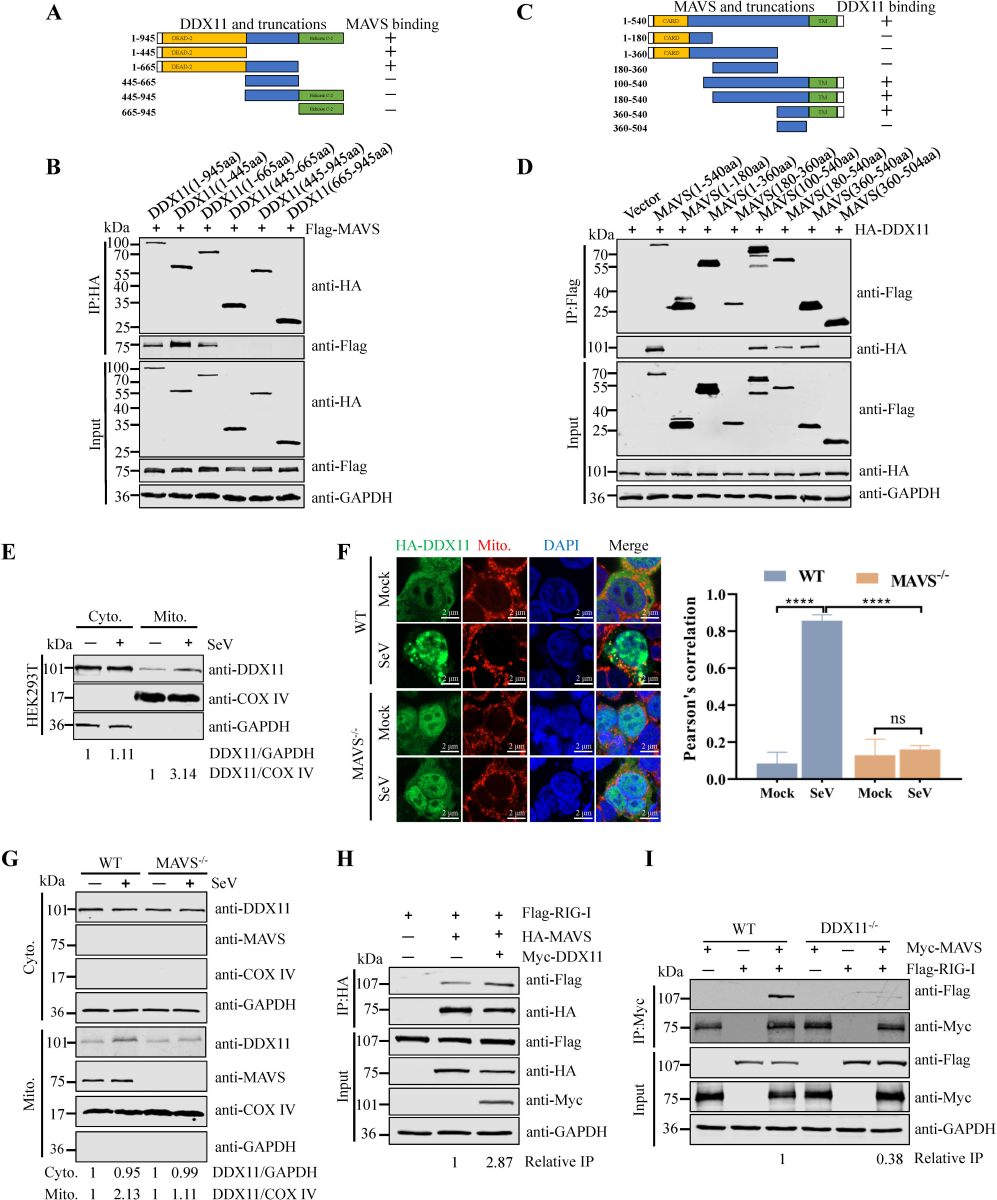
DDX11 promotes RIG-I and MAVS interaction. (**A**) Schematics of full-length DDX11 and its serial-truncated mutants. (**B**) Identification of the domain of DDX11 required for interaction with MAVS. HEK293T cells were transfected with Flag-MAVS and serial-truncated DDX11. Cell lysates were collected for Co-IP and immunoblot analyses. (**C**) Schematics of full-length MAVS and its serial-truncated mutants. (**D**) Identification of the domain of MAVS required for interaction with DDX11. (**E**) The protein levels of DDX11 increase in mitochondria after SeV infection. HEK293T cells were infected SeV for 12 h, and cells were collected for mitochondrial and cytoplasmic extraction. Proteins obtained from the mitochondrial and cytoplasmic fractions were analyzed by western blotting with the indicated antibodies. Calculated band density values for DDX11/GAPDH and DDX11/COX IV; the values of the control group are standardized to one. (**F**) Localization of DDX11 and mitochondria using confocal microscopy in WT HEK293T and MAVS^−/−^ HEK293T cells. WT HEK293T and MAVS^−/−^ HEK293T cells were transfected with HA-DDX11 for 24 h and then infected with SeV. The mitochondria are colored red and DDX11 protein is colored green. The position of the nucleus was visualized by staining with DAPI (blue); merged images are also presented (Bar: 2 µm). Co-localization of DDX11 and mitochondria was performed using the Pearson correlation coefficient. (**G**) WT HEK293T and MAVS^−/−^ HEK293T cells were infected with SeV. The cells were collected for mitochondrial and cytoplasmic extraction. Protein from these fractions was analyzed by western blotting with the indicated antibodies. Calculated band densities for DDX11/GAPDH and DDX11/COX IV; the values of the control group are standardized to one. (**H and I**) DDX11 enhances the interaction of RIG-I with MAVS. (**H**) Co-IP and western blotting analyses of HEK293T cells transfected with Flag-RIG-I, HA-MAVS, and Myc-DDX11 or empty vector are shown. The band density values for interacting protein/IP protein in the IP samples are presented below the corresponding figure. (**I**) WT HEK293T and DDX11^−/−^ HEK293T cells were transfected with Flag-RIG-I and Myc-MAVS and then infected with SeV for 12 h. The interaction of RIG-I with MAVS was analyzed by Co-IP and western blotting. The band density values for interacting protein/IP protein in the IP samples are presented below the corresponding figure.

Mitochondria are pivotal platforms for intracellular antiviral signaling. Numerous mitochondrial proteins play important roles in modulating antiviral signaling. As shown above, DDX11 interacted with the RIG-I and MAVS and promoted RIG-I-MAVS-mediated signaling. To identify the critical region of interaction between DDX11 and MAVS, we constructed a series of truncated mutants of DDX11 and MAVS. MAVS comprises 540 aa containing a CARD, proline-rich domain, and transmembrane (TM) domain (28). Co-IP domain mapping experiments indicated that the N-terminus (1–445 aa) of DDX11 and the C-terminal TM domain of MAVS were required for their interaction (Fig. 6A through D). Because MAVS was localized in mitochondria and interacts with DDX11, we further isolated the mitochondrial fraction and observed increased localization of DDX11 to mitochondria in HEK293T cells after infection with SeV (Fig. 6E). In addition, confocal microscopy revealed the cytoplasmic and nuclear distribution of ectopically expressed DDX11 (green) in HEK293T cells, with minimal colocalization with mitochondria observed prior to SeV infection. Upon SeV infection, a marked increase in the co-localization of DDX11 with mitochondria was evident (Fig. 6F). However, co-localization of DDX11 with mitochondria was not observed in MAVS^−/−^ HEK293T cells, suggesting that the localization of DDX11 to mitochondria upon viral infection was dependent on MAVS (Fig. 6F). Furthermore, the cytoplasmic and mitochondrial fractions of both WT and MAVS^−/−^ HEK293T cells were separated. Western blotting analysis revealed an increase in DDX11 levels in the mitochondria of WT HEK293T cells upon SeV stimulation (Fig. 6G). Conversely, no significant change was observed in the mitochondria of MAVS^−/−^ HEK293T cells (Fig. 6G). To further characterize how DDX11 transduced the antiviral signals upon interaction with MAVS, we explored the relationship between DDX11, RIG-I, and MAVS. As shown in Fig. 6H, Co-IP results showed that overexpression of DDX11 promoted the interaction of RIG-I-MAVS. Notably, knockout of DDX11 decreased the interaction among RIG-I and MAVS upon infection with SeV (Fig. 6I). The collective findings revealed that DDX11 was dependent on MAVS to accumulate in mitochondria after viral infection, which enhanced the formation of the RIG-I-MAVS complex.

### DDX11 aa 445–665 inhibit RNA virus replication

**Fig 7:**
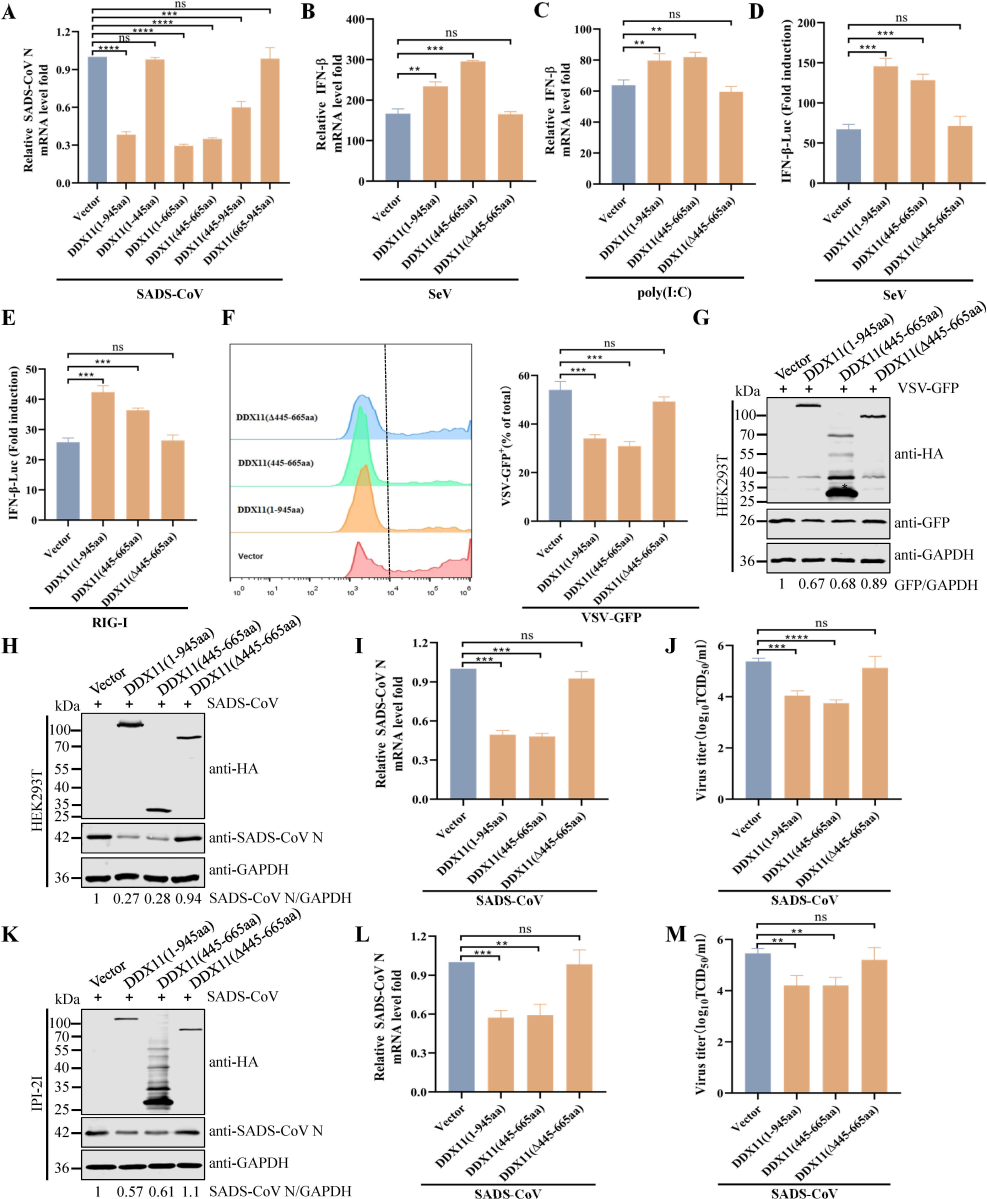
Amino acids 445–665 of DDX11 inhibit RNA virus replication. (**A**) Screening for key domains of DDX11 that inhibit SADS-CoV replication. HEK293T cells were transfected with an empty vector or plasmids expressing the DDX11 truncations for 24 h and then infected with SADS-CoV for 24 h. Cellular RNA was extracted and analyzed by qRT-PCR to detect mRNA levels of SADS-CoV N. (**B and C**) Amino acids 445–665 of DDX11 increase the transcription of IFN-β after SeV infection and poly(I:C) transfection in HEK293T cells. The transcription levels of IFN-β in HEK293T cells that transfected with empty vector or DDX11 (aa 1–945), DDX11 (aa 445–665), or DDX11 (aa Δ445–665) expression plasmids and stimulated with SeV (**B**) or poly(I:C) (**C**) were evaluated by qRT-PCR. (**D**) Amino acids 445–665 of DDX11 increase the SeV-mediated IFN-β promoter activity in HEK293T cells. HEK293T cells were transfected with IFN-β-Luc, pRL-TK, and empty vector or DDX11 (aa 1–945), DDX11 (aa 445–665), or DDX11 (aa Δ445–665) expression plasmids for 24 h. Cells were infected with SeV for 12 h, and luciferase activity was quantified utilizing a dual luciferase reporter assay system. (**E**) Amino acids 445–665 of DDX11 increase the activation of IFN-β luciferase reporters mediated by RIG-I. HEK293T cells were co-transfected with Flag-RIG-I, IFN-β-Luc, pRL-TK plasmid, and empty vector or DDX11 (aa 1–945), DDX11 (aa 445–665), or DDX11 (aa Δ445–665) expression plasmids for 24 h. Luciferase activity was quantified utilizing a dual luciferase reporter assay system. (**F and G**) Amino acids 445–665 of DDX11 inhibit VSV-GFP replication. HEK293T cells were transfected with plasmids expressing DDX11 (aa 1–945), DDX11 (aa 445–665), or DDX11 (aa Δ445–665) for 24 h and then infected with VSV-GFP for 12 h. (**F**) Fluorescence was analyzed by flow cytometry. (**G**) Protein expression of full-length DDX11, DDX11 truncations, and GFP detected by western blotting. Calculated band densities for GFP/GAPDH; the values of the empty vector-transfected group are standardized to one. (**H–M**) Amino acids 445–665 of DDX11 inhibit SADS-CoV replication. HEK293T or IPI-2I cells were transfected with plasmids expressing DDX11 (aa 1–945), DDX11 (aa 445–665), or DDX11 (aa Δ445–665) for 24 h and then infected with SADS-CoV for 24 h. (**H and K**) Protein expression of full-length DDX11, DDX11 truncations, and SADS-CoV N detected by western blotting. Calculated band densities for SADS-CoV N/GAPDH; the values of the empty vector-transfected group are standardized to one. (**I and L**) qRT-PCR assessed transcriptional levels of SADS-CoV N protein. (**J and M**) The SADS-CoV TCID_50_ in the supernatants was titrated on Vero E6 cells. Means and SD (error bars) of three independent experiments are indicated (*P < 0.05; **P < 0.01; ***P < 0.001; ****P < 0.0001; ns, not significant).

To delineate the specific region of DDX11 responsible for inhibiting SADS-CoV replication, HEK293T cells were transfected with various truncations of DDX11. The qRT-PCR analysis showed that the minimal domain of DDX11 capable of suppressing SADS-CoV replication spanned aa 445–665 (Fig. 7A). The qRT-PCR analysis also indicated that overexpressed DDX11 (1–945 aa) and DDX11 (445–665 aa), but not DDX11 (Δ445–665 aa; deletion of the 445–665 amino acids of DDX11), significantly increased the SeV- or poly(I:C)-induced transcription of IFN-β (Fig. 7B and C). Consistently, overexpression of DDX11 (1–945 aa) and DDX11 (445–665 aa) increased SeV- and RIG-I-induced IFN-β promoter activation (Fig. 7D and E). We next investigated whether aa 445–665 of DDX11 might regulate VSV-GFP infection and proliferation. Flow cytometry and western blotting showed that DDX11 (1–945 aa) and DDX11 (445–665 aa) plasmid-transfected HEK293T cells inhibited VSV-GFP replication (Fig. 7F and G), compared with vector- and DDX11(Δ445–665 aa)-transfected cells. Similarly, western blotting, qRT-PCR, and TCID_50_ results showed that overexpression of DDX11 (1–945aa) and DDX11 (445–665aa), but not DDX11(Δ445–665aa), inhibited SADS-CoV replication in HEK293T and IPI-2I cells (Fig. 7H through M). Collectively, these data indicated that aa 445–665 of DDX11 activated the RIG-I-dependent IFN signaling pathway, thereby inhibiting VSV-GFP and SADS-CoV replication.

## DISCUSSION

Extensive functional and structural studies performed in recent years have unequivocally demonstrated the selective binding of viral RNA ligands by RLRs, leading to the initiation of downstream signaling cascades (33). Several DExD/H-box helicases have been implicated in innate immunity against viral infections. However, fundamental questions remain regarding the precise regulatory mechanisms by which DExD/H-box helicases modulate innate immunity. The present study provided evidence of a novel role of the previously uncharacterized helicase DDX11 in the regulation of RIG-Ι-MAVS-mediated antiviral response on RNA virus infection. First, DDX11^−/−^ HEK293T cells infected with SADS-CoV displayed higher titers of viral protein and virus (Fig. 1). These findings suggested the involvement of DDX11 in host defense mechanisms. Second, overexpression of DDX11 significantly potentiated SeV- and poly (I:C)-induced expression of IFN-β, ISG56, and CXCL10 mRNA, whereas knockdown or knockout of DDX11 had the opposite effects (Fig. 2 and 3). Third, DDX11 targeted RIG-I and MAVS to activate IFN production (Fig. 4). Fourth, DDX11 bound to nucleic acids through its 445–665 aa domain and promoted the binding of RIG-Ι to nucleic acids (Fig. 5). Finally, DDX11 interacted with MAVS, and this interaction was increased after viral infection. Overexpression of DDX11 increased the association of RIG-Ι with MAVS (Fig. 6). The findings revealed a distinctive molecular mechanism by which DDX11 modulated the antiviral response.

**Fig 8:**
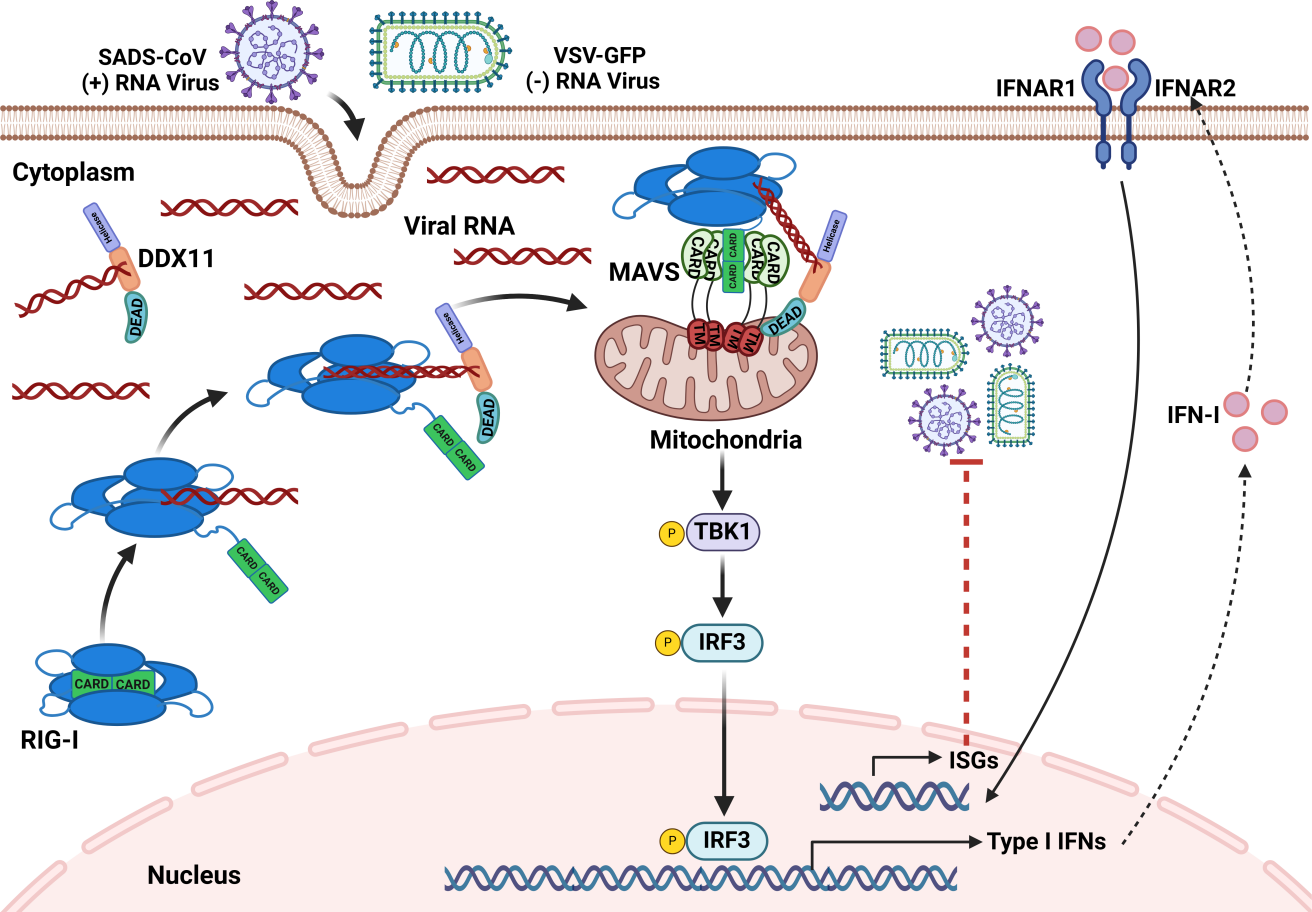
Schematic depiction of the proposed role of DDX11 in antiviral immunity against RNA virus infection. Upon SADS-CoV or VSV-GFP infection, DDX11 functions as an RNA co-sensor to facilitate RIG-I recognition of viral RNA and subsequently relies on MAVS localization in mitochondria to increase RIG-I-MAVS interactions. This activation cascade induces the phosphorylation of TBK1 and IRF3, which subsequently promotes their nuclear translocation and induces IFN production.

The innate immune system employs PRRs in various cellular compartments to recognize the conserved PAMPs of invading pathogens (34). Several of these PRRs have been well characterized, including RIG-Ι. RIG-Ι recognizes viral RNA allowing for a conformational change and triggers the production of IFN and other inflammatory cytokines via interactions with MAVS (35). Activation of MAVS by RIG-I recruits TBK1, which subsequently phosphorylates IRF3 and IRF7, facilitating their translocation to the nucleus (36). This process induces the expression of a myriad of cytokines and chemokines, including IFN (37). Here, we provided several lines of evidence to demonstrate that DDX11 acted as a co-receptor to promote viral RNA binding to RIG-Ι, and the interaction of RIG-Ι and MAVS upon viral infection. This process promotes the phosphorylation of TBK1 and IRF3, which subsequently translocated to the nucleus to activate the IFN-β promoter and stimulate the production of ISGs, thus effectively initiating antiviral immunity. A model for this process is provided in Fig. 8.

The present study provided novel insights into the involvement of DDX11 in the activation of RIG-I-MAVS-mediated innate immunity. The functional role of DDX11 has been unclear. DDX11 is ubiquitously expressed in cells and has been recognized for its involvement in lagging strand DNA replication processes, as well as in maintaining fork structures crucial for establishing cohesion (38). DDX11 has been associated with the development of various cancers, including lung and melanoma cancer (39, 40). Despite the critical roles of DDX11 in DNA replication and the development of cancer, its role in host defense is unknown. In the present study, overexpression of DDX11 restricted SADS-CoV and VSV-GFP infection, whereas knockdown or knockout of DDX11 enhanced SADS-CoV and VSV-GFP replication. These findings indicated the presence of an unidentified mechanism of antiviral activity associated with DDX11, which operated independently of its canonical function. Indeed, there is growing recognition that DExD/H-box helicase family members possess antiviral activity beyond their traditional canonical functions. For instance, DDX10 positively regulates IFN production and inhibits porcine reproductive and respiratory syndrome virus proliferation (41). DDX20 positively modulates the IFN signaling pathway, leading to the inhibition of viral replication, particularly against VSV and herpes simplex virus type I infections (42). Overexpression of DDX23 suppresses foot-and-mouth disease virus RNA synthesis and protein expression (43). Host cellular RNA helicases also regulate severe acute respiratory syndrome-coronavirus-2 (SARS-CoV-2) infection, as evidenced by the necessity of DDX1, DDX5, and DDX6 RNA helicases for SARS-CoV-2 replication. Conversely, DDX21 and MOV10 have been identified as inhibitors of SARS-CoV-2 infection (44). The clarification of this supplementary pathway by which DDX11 affects antiviral responses is expected to provide new perspectives on host-pathogen interactions.

Eukaryotes contain a collective of 59 DExD/H-box helicases, which are categorized into distinct subfamilies that include RIG-I-like, DEAH/RHA, DEAD-box, and Ski2-like (45). Previous studies presented evidence that the involvement of DExD/H-box helicases in antiviral innate immune responses may have a wider scope than previously anticipated. Indeed, recent studies have revealed the participation of DExD/H-box helicases in innate immunity, where they act as co-receptors with RLRs (15, 16, 46–49) or are involved in other pathways (13, 18, 50–52). The extensive involvement of numerous members from this protein superfamily suggests that DExD/H-box helicases play pivotal roles in combating pathogens, in addition to their conventional functions in RNA metabolism (53). An intriguing question arises: why do RLRs like RIG-I necessitate the involvement of multiple factors for the full induction of IFN? One plausible explanation is that, given the status of RIG-I as an ISG with minimal basal expression, there is a critical need for the swift initiation of RLR-mediated signaling pathways. DExD/H-box helicases may fulfill this requirement by augmenting or sustaining PAMP recognition for PRRs (15, 48) or by facilitating the recruitment or redistribution of PRRs to signaling platforms (13, 51, 54). Additionally, these helicases may aid in the identification of PAMPs based on sequence signals, supplementing RIG-I’s recognition of 5′-ppp RNA and short dsRNA (55, 56). The cytoplasm contains substantial quantities of DDX11, while only minimal amounts of RIG-I are detected in resting cells. Consequently, upon viral infection of host cells, viral RNA may first encounter DDX11 before being recognized by RIG-I. Consistent with this hypothesis, the present study provided evidence that DDX11 acted as a co-receptor for the binding of RLR to RNA. Co-IP results indicated that DDX11 interacted with RIG-Ι in uninfected cells and that their interaction was enhanced with the stimulation of RNA virus infection. DDX11 can bind to dsRNA and RIG-Ι via aa 445–665, thereby enhancing the affinity of RIG-I for dsRNA. Consistently, DDX11 deficiency led to decreased binding of RIG-I to dsRNA and hindered their recruitment to the downstream adaptor protein MAVS. Specifically, the removal of aa 445–665 from DDX11 resulted in the loss of its capacity to induce the expression of IFN-β mRNA and activate the IFN-β promoter. Functionally, DDX11 deficiency impaired the signaling mediated by RIG-I and MAVS. Taken together, these findings strongly indicated that DDX11 acted as a crucial co-receptor for RIG-I, facilitating innate immune responses to viral RNA.

Upon virus infection, activated RIG-I participates in downstream signaling pathways by interacting with MAVS. MAVS is located on the outer membrane of the mitochondria, peroxisomes, and the mitochondria-associated membrane, a specialized subdomain of the endoplasmic reticulum recognized as a central site for intracellular innate immune and inflammatory signaling (57). In order to initiate immune signaling, RIG-Ι re-localizes to the mitochondria-associated membrane compartment for the binding of MAVS (57). The results obtained with RIG-I^−/−^ and MAVS^−/−^ HEK293T cells showed that DDX11 could not synergize with SeV in the activation of IFN-β mRNA expression (Fig. 4), which confirmed that DDX11 could positively regulate the RIG-Ι-MAVS pathway. Meanwhile, in addition to acting as a co-receptor for ligand binding of RIG-Ι, DDX11 could also enhance the interaction of RIG-Ι and MAVS and activation of RIG-Ι-MAVS-mediated signaling. Further experiments demonstrated that the DEAD domain (1–665 aa) of DDX11 was responsible for MAVS binding. Thus, it appeared that DDX11 specifically interacted with RIG-Ι through aa 445–665 and binds to MAVS through its N-terminal DEAD domain, thus bringing RIG-Ι and MAVS together. Further exploration is warranted to elucidate the specific domains of DDX11 responsible for its interaction with RIG-I and MAVS. Additionally, a comprehensive analysis and investigation into the collaborative mechanisms of these proteins are essential for understanding how they spatially and temporally regulate MAVS activity.

In summary, our research provided the first evidence that DDX11 was a positive regulator of interferon pathway by targeting RIG-I and MAVS to suppress the replication of SADS-CoV and VSV-GFP. Furthermore, DDX11 acted as a co-receptor to promote viral RNA binding to the RIG-Ι, as well as the interaction of RIG-Ι and MAVS. Collectively, the findings pinpointed the DDX11-RIG-I-MAVS pathway as a pivotal cytosolic nucleic acid-sensing mechanism crucial for fine-tuning the innate immune response. Many reported and unreported helicase family members may play significant roles in regulating the distinctive characteristics of each particular pathway. Comprehensive elucidation of these pathways will contribute to a deeper understanding of diverse infections and immune dysfunctions and will inform the development of innovative therapeutic interventions.

## Data Availability

Data will be made available upon request.

## SUPPLEMENTAL MATERIAL

Fig. S1 mbio.02028-24-s0001.tiff DDX11 knockdown reduces SeV and poly(I:C)-induced IFN response.

Legend mbio.02028-24-s0002.docx Legend for Fig. S1.

Table S1 mbio.02028-24-s0003.docx PCR primer sequences for plasmid construction.

Table S2 mbio.02028-24-s0004.docx The siRNA target sequences used in this study.

Table S3 mbio.02028-24-s0005.docx Primer sequences for sgRNA.

Table S4 mbio.02028-24-s0006.docx Primer sequences for qRT-PCR.

ASM does not own the copyrights to Supplemental Material that may be linked to, or accessed through, an article. The authors have granted ASM a non-exclusive, world-wide license to publish the Supplemental Material files. Please contact the corresponding author directly for reuse.
