# Supplementary figures and images for: Helicase protein DDX11 as a novel antiviral factor promoting RIG-I-MAVS-mediated signaling pathway
Source: mBio. 2024 Oct 29;15(12):e02028-24. doi: 10.1128/mbio.02028-24 (PMC11633105; doi:10.1128/mbio.02028-24)

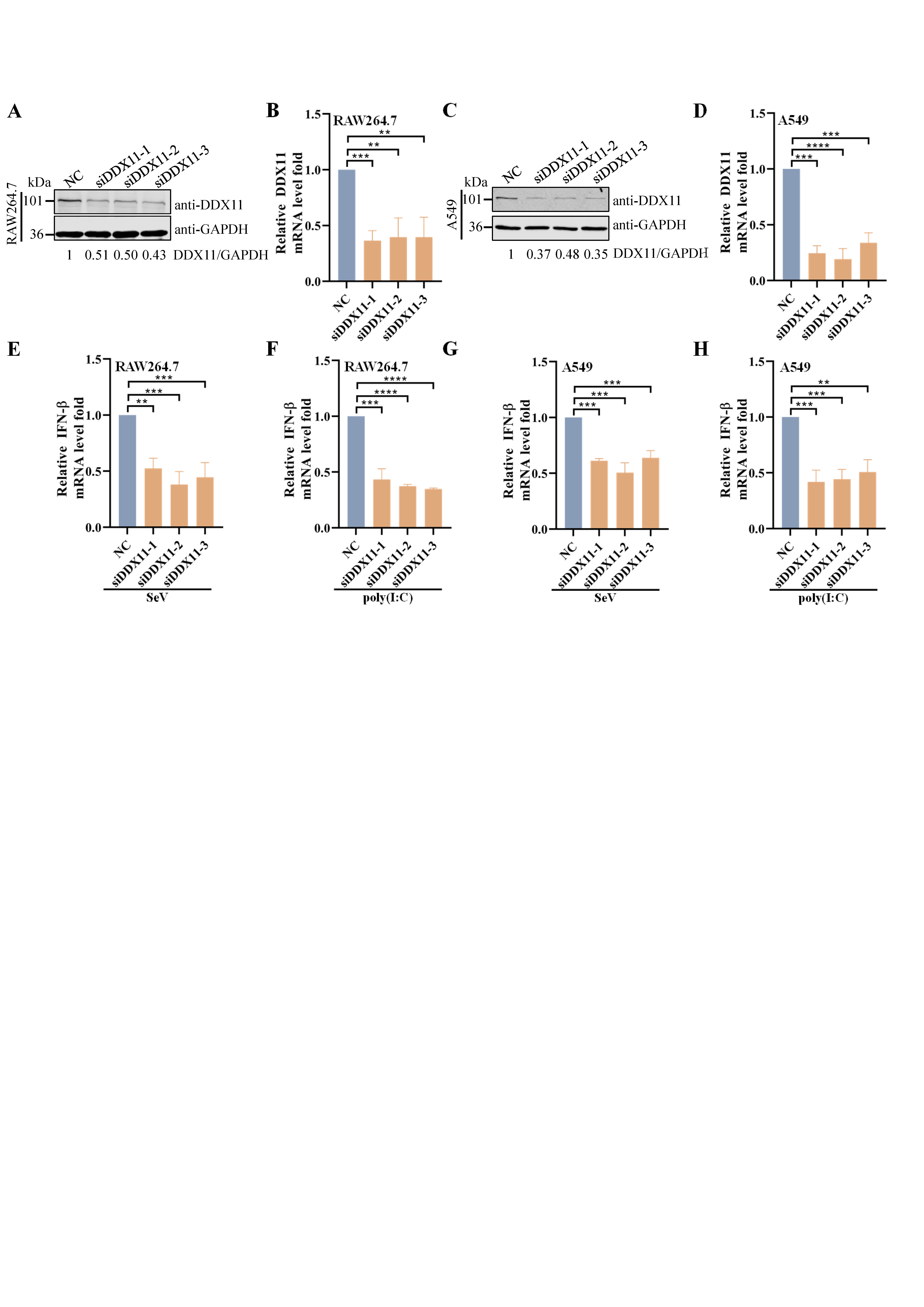

Supplement: Fig. S1 — DDX11 knockdown reduces SeV and poly(I:C)-induced IFN response. [file mbio.02028-24-s0001.tiff]
