# Supplementary material for: Helicase protein DDX11 as a novel antiviral factor promoting RIG-I-MAVS-mediated signaling pathway
Source: mBio. 2024 Oct 29;15(12):e02028-24. doi: 10.1128/mbio.02028-24 (PMC11633105; doi:10.1128/mbio.02028-24)
Supplement: Legend — for Fig. S1. [file mbio.02028-24-s0002.docx]

**FIG. S1.** DDX11 knockdown reduces SeV and poly(I:C)-induced IFN response. RAW264.7 cells or A549 cells were transfected with siDDX11-1, siDDX11-2, siDDX11-3, or siNC (negative control) at 50 nM for 36 h. (**A** and **C**) Protein expression levels of DDX11 analyzed by western blotting. Calculated band densities values for DDX11/GAPDH; the values of siNC group are standardized to one. (**B** and **D**) The mRNA levels of DDX11 determined by qRT-PCR. (**E-H**) RAW264.7 cells or A549 cells were transfected with various siRNAs for 36 h and then infected with SeV (E and G) or stimulated with poly(I:C) for 12 h (F and H). The mRNA levels of IFN-β were determined by qRT-PCR. Means and SD (error bars) of three independent experiments are indicated (^*^*P* < 0.05; ^**^*P* < 0.01; ^***^*P* < 0.001; ^****^*P* < 0.0001; ns, not significant).
