# Supplementary material for: Helicase protein DDX11 as a novel antiviral factor promoting RIG-I-MAVS-mediated signaling pathway
Source: mBio. 2024 Oct 29;15(12):e02028-24. doi: 10.1128/mbio.02028-24 (PMC11633105; doi:10.1128/mbio.02028-24)
Supplement: Table S1 — PCR primer sequences for plasmid construction. [file mbio.02028-24-s0003.docx]

Supplementary Table 1. PCR primer sequences for plasmids construction

| Names | | Sequences (5'-3') | |
| --- | --- | --- | --- |
| HA-DDX11 F | | AGATTACGCTGAATTCATGGCAGAGCTGTACCGGG |  |
| HA-DDX11 R | | GATCTGCTAGCTCGATCATTCAGGATTCCCCCATTTTCTCC |  |
| Myc-DDX11 F | | CCCGAATTCGGTCGACCAATGGCAGAGCTGTACCGG |  |
| Myc-DDX11 R | | GCCGCGGTACCTCGATCATTCAGGATTCCCCCATTTTCTCC |  |
| HA-sDDX11 F | | AATTCGAGCTCATCGGCATGGCTGACAAAACCCAGGAG |  |
| HA-sDDX11 R | | ATTAAGATCTGCTAGTCAGGAAGAGCCACACTTCT |  |
| HA-DDX24 F | | AATTCGAGCTCATCGATATGAAGTTGAAGGACACAAAATCAAGGC |  |
| HA-DDX24 R | | ATTAAGATCTGCTAGTTAATTTGCACTTGTACTTGGCTGTGGCTG |  |
| HA-DDX11-1-445 F | | AATTCGAGCTCATCGATATGGCAGAGCTGTACCGG |  |
| HA-DDX11-1-445 R | | ATTAAGATCTGCTAGTGTCTGTGACAGACTCTGTGTATTGGGA |  |
| HA-DDX11-1-665 F | | AATTCGAGCTCATCGATATGGCAGAGCTGTACCGG |  |
| HA-DDX11-1-665 R | | ATTAAGATCTGCTAGAATGCGACCCACCTCG |  |
| HA-DDX11-445-665 F | | AATTCGAGCTCATCGATACAGGGACGGAGCTGAAGAC |  |
| HA-DDX11-445-665 R | | ATTAAGATCTGCTAGAATGCGACCCACCTCG |  |
| HA-DDX11-445-945 F | | AATTCGAGCTCATCGATACAGGGACGGAGCTGAAGAC |  |
| HA-DDX11-445-945 R | | ATTAAGATCTGCTAGTCATTCAGGATTCCCCCATTTTCTCCT |  |
| HA-DDX11-665-945 F | | AATTCGAGCTCATCGATATTCTCTGTAACCTGTGCGGTG |  |
| HA-DDX11-665-945 R | | ATTAAGATCTGCTAGTCATTCAGGATTCCCCCATTTTCTCCT |  |
| HA-DDX11-Δ445-665F | | ATACACAGAGTCTGTCACAGCTCTGTAACCTGTGCGGTGT |  |
| HA-DDX11-Δ445-665R | | ACACCGCACAGGTTACAGAGCTGTGACAGACTCTGTGTAT |  |
| Flag-MAVS F | | TGACGATGACAAGCTTATGCCGTTTGCTGAAGACAAGAC |  |
| Flag-MAVS R | | CTCTAGAGTCGACTGCTAGTGCAGACGCCGCC |  |
| Flag-MAVS-1-180 F | | TGACGATGACAAGCTTATGCCGTTTGCTGAAGACAAGAC |  |
| Flag-MAVS-1-180 R | | CTCTAGAGTCGACTGCTAGGACTCCAGGGGGCCAC |  |
| Flag-MAVS-1-360 F | | TGACGATGACAAGCTTATGCCGTTTGCTGAAGACAAGAC |  |
| Flag-MAVS-1-360 R | | CTCTAGAGTCGACTGCTATGGCACCATGCCAGCACGG |  |
| Flag-MAVS-180-360 F | | TGACGATGACAAGCTTTCCTCTGACCTGGCAGCC |  |
| Flag-MAVS-180-360 R | | CTCTAGAGTCGACTGCTATGGCACCATGCCAGCACGG |  |
| Flag-MAVS-100-540 F | | TGACGATGACAAGCTTTCGGACCGTCCCCCAGAC |  |
| Flag-MAVS-100-540 R | | CTCTAGAGTCGACTGCTAGTGCAGACGCCGCC |  |
| Flag-MAVS-180-540 F | | TGACGATGACAAGCTTTCCTCCTCTGACCTGGCAG |  |
| Flag-MAVS-180-540 R | | CTCTAGAGTCGACTGCTAGTGCAGACGCCGCC |  |
| Flag-MAVS-360-540 F | | TGACGATGACAAGCTTCCATCCAAAGTGCCTACTAGCAT |  |
| Flag-MAVS-360-540 R | | CTCTAGAGTCGACTGCTAGTGCAGACGCCGCC |  |
| Flag-MAVS-360-504 F | | TGACGATGACAAGCTTCCATCCAAAGTGCCTACTAGCAT |  |
| Flag-MAVS-360-504 R | | CTCTAGAGTCGACTGCTACCTCTCCTGGAACTTCCGG |  |
| Myc-MAVS F | | ATGGAGGCCCGAATTATGCCGTTTGCTGAAGACAAGAC |  |
| Myc-MAVS R | | ATCCCCGCGGCCGCGCTAGTGCAGACGCCGCC |  |
| HA-MAVS F | | AATTCGAGCTCATCGGCATGCCGTTTGCTGAAGACAAG |  |
| HA-MAVS R | | ATTAAGATCTGCTAGCTGGTGCAGACGCCGCC |  |
| tRSA-3'-UTR F | | TGCAGAAAAAAAAAAAACATACAATCCCTAGCTTTGCTTG |  |
| tRSA-3'-UTR R | | GAATTCTTTTTTTTTTTTTTTTGTGTATCACTGTCAAAGAATC |  |
| tRSA-5'-UTR F | | CACTGCAGAAAAAAAAAAAAACATGGGGACTTAAAGATATAATCTA |  |
| tRSA-5'-UTR R | | GAATTCCAATTGGTTGATAGCCATTGTGCACA |  |
|  |  | |  |
