# Supplementary material for: Helicase protein DDX11 as a novel antiviral factor promoting RIG-I-MAVS-mediated signaling pathway
Source: mBio. 2024 Oct 29;15(12):e02028-24. doi: 10.1128/mbio.02028-24 (PMC11633105; doi:10.1128/mbio.02028-24)
Supplement: Table S2 — The siRNA target sequences used in this study. [file mbio.02028-24-s0004.docx]

Supplementary Table 2. The siRNA target sequences used in this study

| Species | Names | Sequences (5'-3') | |
| --- | --- | --- | --- |
| Human | siDDX11-1 | CTCTTGGCTCCGTGACTTT |  |
|  | siDDX11-2 | ACCTGGTGGACCGACTAAA |  |
|  | siDDX11-3 | TGAAAAGCCTAGGTTCTGT |  |
| Mouse | siDDX11-1 | GCGTATTCCAAGTGCATTA |  |
|  | siDDX11-2 | GGAAGCGTTTAAAGGCCAA |  |
|  | siDDX11-3 | GGATGAGATTCTGCTGGAA |  |
| Swine | siDDX11-1 | CACGTGACTAAGATTTACT |  |
|  | siDDX11-2 | AGATCGACAACATCAACTT |  |
|  | siDDX11-3 | CAGAGCAGCCTCAAATTCT |  |
|  |  |  |  |
