# Supplementary material for: Helicase protein DDX11 as a novel antiviral factor promoting RIG-I-MAVS-mediated signaling pathway
Source: mBio. 2024 Oct 29;15(12):e02028-24. doi: 10.1128/mbio.02028-24 (PMC11633105; doi:10.1128/mbio.02028-24)
Supplement: Table S3 — Primer sequences for sgRNA. [file mbio.02028-24-s0005.docx]

Supplementary Table 3. Primer sequences for sgRNA

| Names | Sequences (5'-3') | |
| --- | --- | --- |
| DDX11 sgRNA F | CACCGGGCCCCACAAATAAGACTTA |  |
| DDX11 sgRNA R | AAACTAAGTCTTATTTGTGGGGCCC |  |
| RIG-I sgRNA F | CACCG GAAAAACAACAAGGGCCCAA |  |
| RIG-I sgRNA R | AAACTTGGGCCCTTGTTGTTTTTCC |  |
| MAVS sgRNA F | CACCG ATTGCGGCAGATATACTTAT |  |
| MAVS sgRNA R | AAACATAAGTATATCTGCCGCAATC |  |
|  |  |  |
