# Supplementary material for: Helicase protein DDX11 as a novel antiviral factor promoting RIG-I-MAVS-mediated signaling pathway
Source: mBio. 2024 Oct 29;15(12):e02028-24. doi: 10.1128/mbio.02028-24 (PMC11633105; doi:10.1128/mbio.02028-24)
Supplement: Table S4 — Primer sequences for qRT-PCR. [file mbio.02028-24-s0006.docx]

Supplementary Table 4. Primer sequences for qRT-PCR

| Names | Sequences (5'-3') | |
| --- | --- | --- |
| IFN-β F | TGGGAGGCTTGAATACTGCCTCAA |  |
| IFN-β R | TCCTTGGCCTTCAGGTAATGCAGA |  |
| CXCL10 F | GTGGCATTCAAGGAGTACCTC |  |
| CXCL10 R | TGATGGCCTTCGATTCTGGATT |  |
| ISG56 F | CATACATTTCCACTATGG |  |
| ISG56 R | TACTCCAGGGCTTCATTCA |  |
| hGAPDH F | TCATGACCACAGTCCATGCC |  |
| hGAPDH R | GGATGACCTTGCCCACAGCC |  |
| sGAPDH F | ACCTCCACTACATGGTCTACA |  |
| sGAPDH R | ATGACAAGCTTCCCGTTCTC |  |
| DDX11 F | TGGAACTGGCCCCTTACATGA |  |
| DDX11 R | CTGCACAAACTGAGTAACCCA |  |
| VSV F | GAAAGGGAACTGTGGGATGA |  |
| VSV R | GAACACCTGAGCCTTTGAGC |  |
| SADS-CoV RIP F | ACTACATGTCTGGTGTGGTGG |  |
| SADS-CoV RIP R | TTGCATACGAGGTGTGACGG |  |
| SADS-CoV N F | CCCCTAAACCGGCTCGTAA |  |
| SADS-CoV N R | CAGAATTAGGAACACGCTTCCA |  |
|  |  |  |
